# Revolutionizing treatment for disorders of consciousness: a multidisciplinary review of advancements in deep brain stimulation

**DOI:** 10.1186/s40779-024-00585-w

**Published:** 2024-12-18

**Authors:** Yi Yang, Tian-Qing Cao, Sheng-Hong He, Lu-Chen Wang, Qi-Heng He, Ling-Zhong Fan, Yong-Zhi Huang, Hao-Ran Zhang, Yong Wang, Yuan-Yuan Dang, Nan Wang, Xiao-Ke Chai, Dong Wang, Qiu-Hua Jiang, Xiao-Li Li, Chen Liu, Shou-Yan Wang

**Affiliations:** 1 Department of Neurosurgery, Beijing Tiantan Hospital, Capital Medical University, Beijing, 100070 China; 2 China National Clinical Research Center for Neurological Diseases, Beijing, 100070 China; 3 Innovative Center, Beijing Institute of Brain Disorders, Beijing, 100070 China; 4 Department of Neurosurgery, Chinese Institute for Brain Research, Beijing, 100070 China; 5 Medical Research Council Brain Network Dynamics Unit, Nuffield Department of Clinical Neurosciences, University of Oxford, Oxford, OX3 7BN UK; 6 Institute of Science and Technology for Brain-Inspired Intelligence, Fudan University, Shanghai, 200433 China; 7 National Laboratory of Pattern Recognition, Institute of Automation, Chinese Academy of Sciences, Beijing, 100080 China; 8 Institute of Medical Engineering and Translational Medicine, Tianjin University, Tianjin, 300072 China; 9 State Key Laboratory of Cognitive Neuroscience and Learning, Beijing Normal University, Beijing, 100080 China; 10 Department of Neurosurgery, Chinese PLA General Hospital, Beijing, 100080 China; 11 Department of Neurosurgery, Ganzhou People’s Hospital, Ganzhou, 341000 Jiangxi China; 12 School of Automation Science and Engineering, South China University of Technology, Guangzhou, 510641 China; 13 School of Electrical and Information Engineering, Tianjin University, Tianjin, 300072 China; 14 School of Information Science and Technology, Fudan University, Shanghai, 200433 China

**Keywords:** Deep brain stimulation, Disorders of consciousness, Segmentation of thalamic nuclei, Local field potentials, Computational modeling

## Abstract

Among the existing research on the treatment of disorders of consciousness (DOC), deep brain stimulation (DBS) offers a highly promising therapeutic approach. This comprehensive review documents the historical development of DBS and its role in the treatment of DOC, tracing its progression from an experimental therapy to a detailed modulation approach based on the mesocircuit model hypothesis. The mesocircuit model hypothesis suggests that DOC arises from disruptions in a critical network of brain regions, providing a framework for refining DBS targets. We also discuss the multimodal approaches for assessing patients with DOC, encompassing clinical behavioral scales, electrophysiological assessment, and neuroimaging techniques methods. During the evolution of DOC therapy, the segmentation of central nuclei, the recording of single-neurons, and the analysis of local field potentials have emerged as favorable technical factors that enhance the efficacy of DBS treatment. Advances in computational models have also facilitated a deeper exploration of the neural dynamics associated with DOC, linking neuron-level dynamics with macroscopic behavioral changes. Despite showing promising outcomes, challenges remain in patient selection, precise target localization, and the determination of optimal stimulation parameters. Future research should focus on conducting large-scale controlled studies to delve into the pathophysiological mechanisms of DOC. It is imperative to further elucidate the precise modulatory effects of DBS on thalamo-cortical and cortico-cortical functional connectivity networks. Ultimately, by optimizing neuromodulation strategies, we aim to substantially enhance therapeutic outcomes and greatly expedite the process of consciousness recovery in patients.

## Background

Disorders of consciousness (DOC) arise from severe brain injuries, such as trauma, stroke, or anoxia, leading to profound loss of awareness. DOC is generally classified into two primary states: vegetative state/unresponsive wakefulness syndrome (VS/UWS) and minimally conscious state (MCS) [1]. Patients in VS/UWS are devoid of self-awareness and environmental perception, while those in MCS exhibit intermittent indications of consciousness, such as variable emotional and directional behaviors [2]. In China, the prevalence of DOC approximates one million, with 50,000–100,000 new cases every year, representing a considerable burden on society and families [3, 4]. Positive outcomes remain scarce despite treatment efforts, including audiovisual and tactile stimulation, non-invasive electromagnetic stimulation, and hyperbaric oxygen therapy [5]. The challenge of identifying effective interventions for patients with impaired consciousness persists, highlighting the necessity for advancing research and therapeutic strategies in this crucial field.

Among the primary etiologies of DOC—trauma, stroke, and anoxia—patients with post-traumatic DOC generally demonstrate better short- and long-term survival rates and recovery of consciousness compared to those with non-traumatic causes. Analysis of clinical progress reveals that patients in the VS/UWS recover more slowly than those in the MCS [6, 7]. VS/UWS patients typically show improvements within 1–2 years after the onset, with most regaining some responsiveness but only a minority reaching MCS. In contrast, most MCS patients achieve full consciousness within the first year, with only a few showing diagnostic transitions out of MCS in the subsequent 1–2 years [8]. These findings underscore the need for targeted therapeutic interventions for MCS patients to support the restoration of full consciousness and reduce motor dysfunction.

Recent advancements in neuromodulation techniques hold promise for enhancing consciousness and cognitive function. Deep brain stimulation (DBS) has demonstrated success in treating neurological disorders such as Parkinson’s disease and dystonia, and it also shows potential as a treatment for DOC. One study indicates that DBS can improve synaptic activity in relevant brain structures, regulate arousal, and facilitate cognitive recovery, particularly when targeted at the central thalamus (CT) [9].

**Fig. 1:**
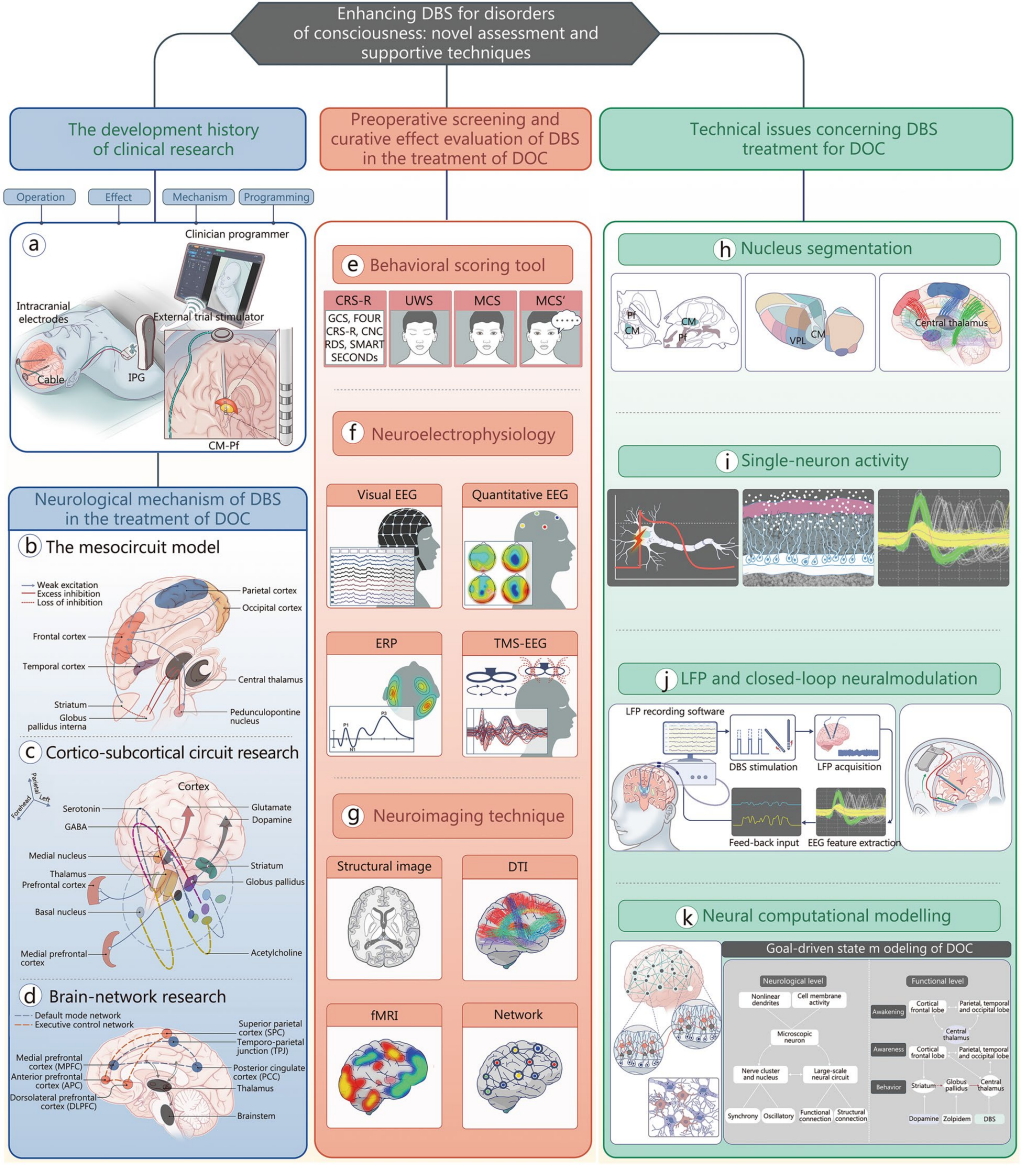
Current progress in DBS for DOC. **a** The CM-Pf is a common DBS target in DOC patients. While the mechanisms of DOC remain partially comprehended, the focus has shifted from isolated brain regions to a comprehensive neural network perspective, spanning macroscopic and microscopic levels. Proposed models for DOC include: the mesocircuit model (**b**), which describes a unifying mechanism based on anterior forebrain mesocircuit downregulation; cortico-subcortical circuit research (**c**), extending from the thalamus to the cortex or striatum, along with neurotransmitters from thalamic nuclei, which offer novel insights into DOC pathophysiology; brain-network research (**d**), which suggests that functional activation within the default mode network (core network) and executive control network (higher-order network) is essential for maintaining consciousness. **e** In clinical settings, diagnostic scales such as GCS, FOUR, CRS-R, CNC, RDS, SMART, and SECONDs are used to stratify consciousness levels in DOC patients. Notably, CRS-R can distinguish between MCS and versus patients. The multimodal approach to DOC diagnosis includes: neuroelectrophysiology (**f**) and neuroimaging technique (**g)**, providing critical insights into cortical and thalamic damage as well as residual consciousness. Here, reduced thalamocortical and thalamostriatal outflow, due to diminished input to striatal MSNs, leads to a failure to reach the discharge threshold. This results in the loss of active inhibition from the striatum to the globus pallidus internus, which typically exerts inhibitory control over its targets. Technological advancements have furthered the treatment of DOC. **h** Thalamic nuclear segmentation reveals the distribution of thalamic subnuclei at an individual level, which is essential for understanding thalamic integrity and guiding personalized DBS interventions. **i** Spike detection techniques identify single-neuron activity, extracting action potentials and firing rates to distinguish thalamic nuclei, assist in the localization of CT-DBS, and assess CT activity across consciousness levels. **j** LFP reflects low-frequency neural discharges, with unique rhythmic patterns indicative of neuronal activity or disease-related functions. Furthermore, in some exploratory studies, the characteristic alterations of LFP are monitored in real-time via implanted targets. Based on these LFP signatures, efficient and energy-saving adaptive closed-loop neural modulations are being designed. **k** Multi-level modeling, integrating microscopic neurons, mesoscopic neural clusters, and circuits into macroscopic brain states, enables the virtual reconstruction of DOC patient networks, providing valuable insights into their complex neural architecture. CT central thalamus, DBS deep brain stimulation, DOC disorders of consciousness, UWS unresponsive wakefulness syndrome, MCS minimally conscious state, MCS’ emergence from minimally conscious state, DTI diffusion tensor imaging, fMRI functional MRI, EEG electroencephalography, ERP event-related potential, TMS transcranial magnetic stimulation, GABA Gamma-Aminobutyric Acid, VS vegetative state, CM-Pf centromedian-parafascicular complex, LFP local field potentials, GCS Glasgow coma scale, FOUR full outline of unresponsiveness, CRS-R coma recovery scale-revised, CNC coma/near coma, SMART sensory modality assessment technique, RDS Rappaport disability rating scale, SECONDS simplified evaluation of consciousness disorders, IPG implantable pulse generator

A notable advantage of neuromodulation lies in its precision in targeting specific brain regions and networks, which can markedly enhance network activity and expedite functional recovery in DOC patients [10, 11]. This review investigates the role of DBS in DOC treatment, examining its historical development, techniques (Fig. 1a), mechanisms (Fig. 1b-d), clinical evaluation methods (Fig. 1e-g), and technological progress (Fig. 1h-k). Through a thorough examination of existing research, this review aims to highlight DBS’s therapeutic benefits, particularly its potential to elevate consciousness and support neural circuit recovery in DOC patients.

The application of DBS in the management of DOC has garnered significant interest among researchers. Studies concentrating on enhancing arousal through DBS frequently target the centromedian-parafascicular complex (CM-Pf), CT, and globus pallidus internus (GPi). Precise localization of target nuclei is essential for successful surgery, and histological or magnetic resonance imaging (MRI)-based atlases for thalamic segmentation improve the accuracy of nucleus localization. Assessing the rhythmicity and connectivity of local field potential (LFP) within nucleus clusters assists in evaluating the therapeutic efficacy of DBS.

Modeling at the neuronal, mesoscopic, and brain state levels provides a comprehensive view of the pathophysiological mechanisms underlying DOC, enabling the analysis of oscillatory states related to arousal, perception, and motor function in DOC patients. These aspects are elaborated upon in subsequent sections.

## History of DBS in treating DOC

In 1949, Moruzzi et al. [12] carried out experiments linking electrical stimulation of the thalamus or midbrain to arousal in the forebrain. Building upon this, Mclardy et al. [13] observed in coma patients that deep brain electrode stimulation could elicit certain neuropathological changes in 1968. Hassler et al. [14] applied DBS to patients with DOC in 1969, targeting the basal part of the right pallidum and the left latero-polar nucleus of the thalamus. They observed notable improvements in arousal and temporary behavioral enhancements, which lasted only 19 d. In 1979, Sturm et al. [15] demonstrated improved communicative behavior in DOC patients following DBS, reinforcing its therapeutic potential. With the increasing understanding of DOC and DBS, applications have expanded. Targeting the CT with DBS (CT-DBS) enhanced patients’ environmental responsiveness and motor function, underscoring its benefits [16].

In the 1980s, clinical studies conducted in Japan on MCS and VS/UWS patients disclosed that DBS targeting the CM-Pf complex yielded substantial improvements: some MCS patients regained communication abilities, and one showed promising recovery (Fig. 1a) [17, 18]. Electroencephalography (EEG) recordings indicated increasing reactivity over time, and cerebrospinal fluid transmitter levels verified the effectiveness of DBS.

The 1990s marked significant progress in implantable neurostimulators, facilitating research on the long-term effects of DBS in DOC patients [19, 20]. Japanese studies were the first to report this advancement, with Yamamoto et al. [21, 22] conducting an extensive study involving 128 DOC patients, including 21 VS patients receiving DBS in the midbrain reticular formation or CM-Pf complex. Among them, 8 exhibited EEG desynchronization and specific brainstem responses. Of the 21 MCS patients treated with CM-Pf DBS, 5 showed clinical improvement [21, 22]. Continuous DBS enhanced awareness and behavior, with some patients regaining communication skills and reintegrating into family life [17].

In 2007, Schiff et al. [9] conducted a clinical trial administering CT-DBS to a patient with post-traumatic MCS persisting for 6 years. The results demonstrated that CT-DBS effectively enhanced awareness, suggesting its potential to counteract frontal lobe arousal deficits after brain injury. The “central thalamic microcircuit” hypothesis was proposed to explain consciousness impairment in DOC patients [23].

The initial DBS studies emphasized its variable efficacy, depending on specific circumstances [24, 25]. In 2016, Magrassi et al. [25] reported positive outcomes in patients undergoing chronic stimulation for 18–48 months. These patients exhibited enhanced θ and γ EEG power spectra and improved coma recovery scale-revised (CRS-R) scores (CRS-R: Developed by the JFK Johnson Rehabilitation Institute at JFK Medical Center, USA, to differentiate subtle neurobehavioral functions and monitor consciousness recovery). The severity of limb spasticity and the frequency/severity of pathological movement were also reduced. Subsequent studies with larger sample sizes further evaluated DBS’s efficacy [26–33]. Meanwhile, Chudy et al. [34] found that the 10-year single-center study of 32 DOC patients treated in the CM-Pf nucleus reported a 21.9% improvement rate of consciousness. Yang et al. [32] revealed that 37 patients undergoing bilateral monopolar stimulation at 100 Hz achieved a 32.4% improvement of consciousness within 1 year, significantly higher than the 4.3% of the conservative group. DBS effects were particularly superior in MCS patients. A predictive nomogram based on various factors confirmed DBS’s potential in DOC treatment. Despite the limited number of cases, two additional clinical observations have also demonstrated that stimulation at 100 Hz can induce an elevation in the level of consciousness [30, 33].

**Fig. 2:**
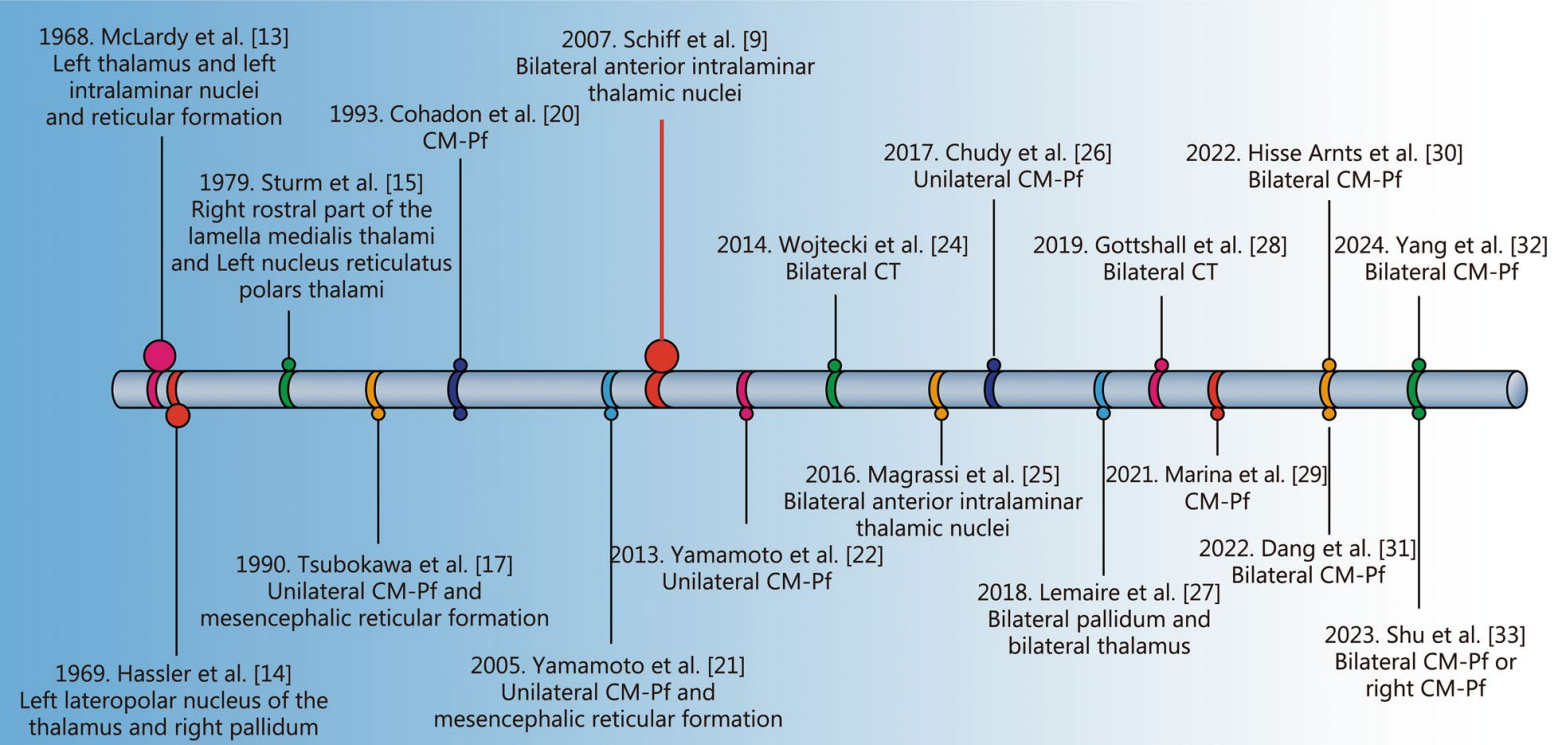
A chronological timeline depicting the involvement of various research and their stimulation targets (including CT and CM-Pf) in the development of DBS therapy for DOC. Since the 1960s, DBS has been investigated for its potential therapeutic efficacy in patients with DOC. Early observations made by Mclardy et al. [13] and Hassler et al. [14] demonstrated temporary improvements in arousal and behavior. As understanding grew, targeted DBS of the centromedian-parafascicular (CM-Pf) complex enhanced environmental responsiveness and motor function. Japanese studies during the 1980s and 1990s further confirmed its efficacy, with clinical improvements and EEG changes reported [17, 18, 21, 22]. Schiff et al. [9] demonstrated the effectiveness of CT-DBS in elevating awareness in post-traumatic MCS patients. Recent studies, inclusive of those by Magrassi et al. [25] and Chudy et al. [26], have shown positive outcomes in patients undergoing chronic stimulation, featuring enhanced EEG power spectra and improved CRS-R scores. Yang et al. [32] found that bilateral monopolar stimulation at 100 Hz achieved remarkable consciousness improvement rates, particularly in MCS patients, further supporting the potential of DBS in DOC treatment. CT central thalamus, CM-Pf centromedian-parafascicular complex, DBS deep brain stimulation, DOC disorders of consciousness, CRS-R coma recovery scale-revised

A recent study by Schiff et al. [35] disclosed that 5 patients with moderate to severe traumatic brain injury (TBI) presented improved executive and cognitive abilities following DBS to the central lateral (CL) thalamic nucleus and the medial dorsal tegmental tract. Their findings support the central thalamic microcircuit hypothesis, suggesting that inadequate CL activation leads to reduced frontal lobe network function after injury. The overall efficacy of DBS for DOC patients was 40% (58/145, based on clinical behavior scale improvements). Among the studies, Tsubokawa et al. [17] reported the highest efficacy at 50% (4/8), while Chudy et al. [26] observed the lowest at 28.6% (4/14), excluding case studies and retrospective analyses (Fig. 2, Table 1).

**Table 1:** Overview of patient reports of DBS for DOC

Reference	Sample/Sex/Age (year)	Etiology	Diagnosis	Time from initial injury to DBS (month)	Brain targets	Follow-up after surgery (month)	Outcome
McLardy et al. [13]	1/male/19	TBI	Coma vigilans	7	Left thalamus and left midbrain for intralaminar nuclei and reticular formation	24	Move left hand; No change in consciousness and died 24 months after surgery
Hassler et al. [14]	1/male/26	TBI	Apallic syndrome or coma vigil	5	Basal portion of the left lateropolar nucleus of the thalamus; Basal part of the right pallidum	< 1	Improvement of consciousness; Spontaneous movements of the left limbs; Unintelligible vocalization
Sturm et al. [15]	1/male/68	Outcome of operation for aneurysm, probably ischemia of the brain stem	Subcoma with unconsciousness	< 1	Right rostral part of the lamella medialis thalami; Left nucleus reticulatus polars thalami	2	Partial and temporary limited improvement; After 2 months the patient died from pneumonia
Tsubokawa et al. [17]	5/males/24, 43, 44, 48, 75; 3/females/41, 41, 74	4 TBI; 3 vascular; 1 anoxic	PVS (PCS 2–4)	> 6	6 non-specific thalamic nucleus (CM-Pf); 2 nucleus cuneiformis in mesencephalic reticular formation; Unilateral	12	3 full recovery (PCS 8–9); 1 incomplete recovery (PCS 7); 3 no recovery (PCS 3–5)
Hosobuchi & Yingling [19]	1/male/23	Anoxic (ischemia)	Apallic state, GCS < 6	8	NA	10	Improvement; Oral feeding possible; Have respond to verbal commands; Not shake or nod the head on yes/no questions
Cohadon & Richer [20]	25/patients/NA	NA	VS	> 3	CM-Pf	12—144	1 improvement to moderate disability (GOS); 10 improvements to severe disability (GOS); 2 died; 12 no recovery
Yamamoto et al. [21]	4/females/19, 41, 58, 59; 3/males/30, 43, 75; 1/person/NA	6 vascular; 2 TBI	PVS (PCS 2–5)	3–6	19 CM-Pf; 2 mesencephalic reticular formation; Unilateral, less injured side	120	All 8 recovered: 7 are bedridden (PCS 8–10), 1 is able to live in a wheelchair
	7/males/29, 30, 42, 44, 48, 49, 56; 6/females/30, 39, 41, 44, 61, 74	7 TBI; 3 vascular; 3 anoxic					All 13 no recovery (PCS 3–7)
Schiff et al. [9]	1/male/38	TBI (closed head injury)	MCS	78	Bilateral, anterior intralaminar thalamic nuclei and adjacent paralaminar regions	24	CRS-R subscales, various improvements (arousal, motor and communication as primary measures); Restoration of communication (interact consistently and meaningfully)
Katayama et al. [18], Yamamoto et al. [22]	5/patients/18—47 (mean: 33.5 ± 14.3)	3 TBI, 2 vascular	MCS	3–6	5 CM-Pf, unilateral, less injured side	120	All 5 recovered, live at home with family; Severe disabled condition (GOS); Need wheelchair, 4/5 patients not operate wheelchair by themselves
Wojtecki et al. [24]	1/female/38	TBI (closed head injury)	DOC (GCS 4)	84	Bilateral, CT (internal medullary lamina and the nuclei reticularis thalami)	NA	No recovery (yet); Patient had increased brain activity on response to her children’ voice
Magrassi et al. [25]	1/male/58	TBI	MCS (CRS-R 14)	28	Bilateral, anterior intralaminar thalamic nuclei and adjacent paralaminar regions	18	CRS-R 15
	1/male/23	TBI	VS/UWS (CRS-R 8)	34		60	CRS-R 11
	1/male/29	TBI	VS/UWS (CRS-R 6)	96		59	CRS-R 9
Chudy et al. [26]	1/male/17	Anoxic (CA)	MCS (C/NC 2.0/1)	2	Unilateral CM-Pf (preferentially on left side, if too damaged right side)	60	C/NC 0, aware; Regained consciousness; Largely independent
	1/male/23	Anoxic (CA)	MCS (C/NC 1.8/1)	2		57	C/NC 0, aware; Regained consciousness; Largely independent; Still experiences short term memory impairment and emotional regression
	1/female/15	TBI	MCS (C/NC 1.6/1)	11		51	C/NC 0, aware; Regained consciousness; Has a severe left side hemiparesis and needs assistance in everyday life; Needs wheelchair
	7/males/17, 17, 20, 25, 34, 43, 59; 4/females/16, 28, 39, 49	3 TBI; 8 anoxic (CA)	1 MCS 10 VS	3—138		38—59	1 VS improved to MCS; 3 died; 7 no recovery
Lemaire et al. [27]	1/male/32	TBI	UWS (CRS-R 6.1; After surgery before CT-DBS ON)	146	Right pallidum; Bilateral thalamus	8	CRS-R 8.4 (41 average)
	1/female/62	Hemorrhagic strokes	MCS (CRS-R 9.6; After surgery before CT-DBS ON)	14	Bilateral pallidum; bilateral thalamus		CRS-R 9.5 (34 average)
	1/male/24	TBI	MCS (CRS-R 11.7; After surgery before CT-DBS ON)	37			CRS-R 13.8 (40 average)
	1/female/22	TBI	MCS (CRS-R 4.8; After surgery before CT-DBS ON)	48			CRS-R 4.3 (28 average)
	1/female/47	Hemorrhagic strokes	MCS (CRS-R 4.2; After surgery before CT-DBS ON)	27			CRS-R 3.0 (27 average)
Gottshall et al. [28]	1/male/17	TBI	MCS (CRS-R 11.8; Average; After surgery before CT-DBS ON)	257	Bilateral CT (the sensory relay nucleus of the thalamus on both the right and left hemispheres)	96.5	MCS; CRS-R 11.8 average; there was no change in CRS-R scores between active CT-DBS time points; change in sleep pattern recorded via EEG
Marina et al. [29] (4 patients were the same as Chudy et al. [26])	1/female/18	TBI	UWS (C/NC, 2.6/2)	14	CM-Pf (preferentially on left side, but, if too damaged right side)	23	C/NC 0, aware; Regained consciousness, has a severe left side Hemiparesis and needs assistance in everyday life; Needs wheelchair
Hisse Arnts et al. [30]	1/female/38	TBI	MCS (CRS-R 9–14)	96	Bilateral, CM-Pf	24	CRS-R 9–12; Increased arousal, visual pursuit, return of swallowing, and reduction of spasticity
Dang et al. [31]	5/males/25, 52, 35, 49, 31; 4/females/35, 45, 11, 26	4 TBI; 3 vascular; 2 anoxic	MCS (CRS-R 9–14)	6–12 months	Bilateral, CM-Pf	6	The CRS-R scores of P4 from 9 to 12, 8 to 11 for P7, and 9 to 16 for P9. EEGs show that the brain functional connectivity of P4, P7 and P9 significantly improved after DBS
Yang et al. [32]	23/males/mean: 26.0–55.5; 14/females/mean: 26.0–55.5	8 trauma; 10 anoxia; 19 stroke	13 MCS; 24 VS/UWS	3–5 months 25(67.6%); 6–11 months 8 (21.6%); ≥ 12 months 4 (10.8)	Bilateral, CM-Pf	12	12 patients (10 in MCS; 2 in VS/UWS) improve in consciousness at 1 year (more than 3 points)
Shu et al. [33]	7/males/48, 37, 60, 46, 61, 53, 55; 3/females/72, 75, 69	4 trauma; 4 hemorrhage; 2 anoxic	2 MCS; 8 VS	3–18 months	4 patients right CM-Pf nuclei; 6 patients bilateral CM-Pf nuclei	1	11 patients showed increased CRS-R scores; P3, P4, and P6 changed from VS to MCS

NA, not available; CA, cardiac arrest; CT, central thalamus; CM-Pf, centre median parafascicular complex; CRS-R, coma recovery scale-revised; C/NC, coma/near coma scale; DBS, deep brain stimulation; DOC, disorders of consciousness; DT, stimulation during day time; EEG, Electroencephalogram; MCS, minimally conscious state; GCS, Glasgow coma scale; GOS, Glasgow outcome scale; PCS, prolonged coma scale; PVS, persistent vegetative state; TBI, traumatic brain injury; UWS, unresponsive wakefulness syndrome; VS, vegetative state

The current absence of globally recognized clinical strategies for DBS in DOC patients stems from the paucity of published randomized controlled trials, which has led to hesitation within the clinical community. Although previous clinical studies on DBS for DOC have shown promising outcomes, the scarcity of large-scale randomized trials and standardized protocols remains a significant obstacle. Promoting DBS as a treatment option for DOC patients necessitates further research, a deeper understanding of DOC pathophysiology, and careful ethical deliberation.

### The mesocircuit model

The theoretical foundation for employing DBS to treat DOC is based on the mesocircuit hypothesis proposed by Schiff et al. [23] in 2010. This hypothesis contends that the CT is crucial in the circuit responsible for consciousness, acting as a relay for cortical commands and sensory signals (Fig. 1b). Through stimulating CT neurons, DBS influences both the thalamus-cortex and thalamus-striatum pathways, facilitating the regulation of abnormal brain metabolic activity [10, 36].

The CT plays a distinctive role in supporting executive functions in the forebrain, regulating arousal by receiving input from the brainstem/basal forebrain arousal system and the frontal cortical system (Fig. 1c). Severe brain injury disrupts neuronal connectivity, leading to circuit dysfunction, reduced synaptic excitability, and decreased excitatory neurotransmission. At the neuronal level, medium spiny neurons (MSNs) in the striatum are essential for maintaining excitability in the anterior forebrain through inhibitory projections to the globus pallidus (GP), which inhibits CT function [23, 37]. Activation of MSNs suppresses the CT, thereby promoting thalamocortical afferents. Central thalamic neurons have extensive connections with the striatum and regulate its background activity. Cases of diffuse brain injury exhibit decreased neural connectivity between the striatum, CT, and frontal cortex, significantly reducing MSN output [23, 38].

Concerning the effects of DBS on neural cells within the targeted nuclei, early experimental evidence supports the hypothesis that DBS inhibits neuronal activity at the stimulation site, and somatic inhibition emerges through several mechanisms. Firstly, high-frequency stimulation in vitro can induce sustained nerve membrane depolarization, deactivating sodium channels and increasing potassium currents to prevent the initiation or propagation of action potential (known as depolarization block) [39]. Additionally, DBS may act via synaptic mechanisms, activating inhibitory presynaptic terminals on cell body afferents, thereby prolonging neuronal conduction time or inducing inhibition [40]. Evidence also suggests that DBS can enhance neural activity by activating axons and dendrites within the stimulation area, increasing the output frequency of action potential [41]. These findings imply a dissociation between somatic and axonal neuronal activity. Computational models indicate that axons and dendrites have lower stimulation thresholds than cell bodies, suggesting that most somatic effects probably result from the propagation of stimulation effects from local membrane arborization rather than the cell body itself [10, 40]. Consequently, DBS may induce action potentials in afferent fibers within the stimulation area, and in some cases, targeting these tracts has become a primary focus of DBS treatment [40, 41].

Following the mesocircuit hypothesis, enhancing the excitatory output of the CT via DBS has the potential to restore normal function in the central circuitry for patients with severe brain injury and DOC by exerting a stable regulatory effect on this circuit. This theory partially explains how DBS applied to CT promotes wakefulness from a clinical perspective [9, 28]. Kundu et al. [38] proposed that the integrity of the reticular structure, cortex, and thalamic circuit is of crucial importance for the effectiveness of DBS in DOC; however, this integrity is not consistently maintained in patients with severe brain injuries.

Studies using rodent models have offered strong evidence that DBS can modulate arousal and overall brain activity. DBS has been demonstrated to enhance arousal and locomotor activity in mice with multiple traumatic injuries [41]. The CL nucleus in the CT is critical in this process, as it receives input from the brainstem reticular activation system and projects to the cortex, particularly its deep layers in the frontoparietal region [42]. In rats, optimized CL activation extensively stimulated forebrain regions, including the frontal cortex, sensorimotor cortex, and striatum, resulting in a remarkable transition from sleep to wakefulness [41]. Similarly, Jordy et al. [36] found that CT-DBS administered to anesthetized rhesus monkeys increased arousal and excitability in specific brain regions, facilitating the restoration of resting-state excitability related to consciousness and sensitivity to auditory stimuli. These studies provide insights into how DBS may improve DOC.

### Brain-network

In the evolving field of consciousness theory, current approaches emphasize identifying explanatory links between neural mechanisms and diverse aspects of consciousness (Fig. 1d). A normative and comprehensive theory of consciousness (TOC) encompasses four primary categories: higher-order theories (HOTs), global workspace theories (GWTs), integrated information theory (IIT), and retrospective and predictive processing theories [43]. These frameworks offer academic viewpoints for observing contemporary states of consciousness. Specifically, GWTs propose that consciousness depends on the integrity of functional or dynamic connections within the frontal-parietal regions, and impairment in these connections can lead to varying degrees of consciousness loss [43]. These theories provide useful perspectives for understanding the origins and mechanisms of consciousness [44].

The recovery of brain activity during DOC rehabilitation is closely associated with the functioning of the anterior forebrain mesocircuit [23, 45, 46] and the frontoparietal network [47, 48]. This network includes the frontal lobe, prefrontal cortex, and a negative feedback loop between the striatum and thalamus, influencing the information flow back to the cortex and striatum [23]. The frontoparietal network consists of two subnetworks: the default mode network (DMN) and the executive control network (ECN). The DMN is anchored by nodes in specific brain areas, such as the medial prefrontal cortex, posterior cingulate gyrus, and precuneus. Studies have shown that DMN changes are correlated with the severity of DOC in patients [47, 49, 50]. Recent research indicates that alterations in the precuneus and inferior parietal lobule contribute to DMN changes due to reduced inhibitory function of the striatum and decreased coupling between the striatum and thalamus [50].

In contrast, the ECN is anchored by nodes in the dorsolateral prefrontal cortex and lateral parietal cortices. Further studies on the interactions of ECN and DMN indicate decreased connectivity across different consciousness states, with dual-task paradigms playing a significant role. At a specific stimulus intensity, only ECN activation enables conscious perception, while DMN activation does not [51–53]. Thibaut et al. [54] found metabolic impairments in both networks in VS/UWS patients, preserved ECN metabolism in MCS patients, and partial recovery in both networks only in EMCS patients. This implied that partial metabolic recovery of these networks may characterize the recovery of MCS. In non-human primate studies, electrical stimulation targeting the CL has been demonstrated to induce arousal from anesthesia-induced coma, providing strong evidence for connectivity between frontoparietal circuits and anterior networks through this brain region [55, 56].

## Evaluation of patients with DOC

### Scale of clinical behavior

The central circuit hypothesis accounts for consciousness loss following brain injury, identifying cortical and subcortical structures, including the CT, as key elements [10, 57]. With a robust theoretical foundation, DBS targeting CT has shown positive effects in clinical studies involving DOC patients. Accurately assessing the neuromodulatory effects of DBS is essential for understanding its neural mechanisms, optimizing technical parameters, and facilitating consciousness recovery in DOC patients (Fig. 1e).

Conventional bedside assessment tools and neurosurgical rating scales, such as the Glasgow coma scale (GCS), have limited utility for monitoring progress in patients with prolonged consciousness disturbances [58]. The Full Outline of UnResponsiveness (FOUR) score is more sensitive than the GCS in detecting different brainstem function levels in the acute phase of severe brain injury [59]. It assesses brainstem reflexes, breathing, and early consciousness indicators, such as visual pursuit, thereby providing improved monitoring for comatose and VS/UWS patients. The Coma/Near Coma (CNC) scale, introduced in 1992, quantifies clinical changes in severely brain-injured, vegetative state patients. It covers traumatic and non-traumatic injuries, with 5 levels ranging from “no coma” to “extreme coma”. Scores are based on 11 items assessing sensory, perceptual, and basic responses, facilitating the identification of patients likely to respond to rehabilitation, with 95 – 97% interrater reliability. Rappaport et al. [60] detail the Disability Rating Scale (DRS), with scores from 0 (complete recovery) to 30 (death), based on phone interviews with patients’ guardians or caretakers, structured to minimize errors and capture behavioral details. The simplicity and accuracy of DRS contribute to its reliability in distinguishing patients’ functional independence [61]. It is more sensitive than the Glasgow outcome scale (GOS) in detecting clinical changes in severe head trauma cases.

Among these scales, the Wessex Head Injury Matrix (WHIM) is recommended, though with certain caveats [62]. It captures changes in VS/UWS patients with post-amnesia, being particularly sensitive to MCS transitions often missed by the GCS, although its reliability and sensitivity are lower than those of the Coma Recovery Scale-Revised (CRS-R) [62]. Other scales, such as the Western Neuro Sensory Stimulation Profile (WNSSP) [63], the Sensory Modality Assessment Technique (SMART) [64], and the Disorders of Consciousness Scale (DOCS) [65], have standardized administration and scoring procedures and are moderately recommended by the American Congress of Rehabilitation Medicine (ACRM) (details in Table 2).

**Table 2:** The application of scales and scores for the assessment of disorders of consciousness

Scale	Content	Estimated time	No. scales (items)	Features of evaluation
GCS	Eye, motor, verbal	5	4 (15)	Not sensitive to differentiate MCS patients
GOS	Physical disability and mental state	5	5 (5)	Main evaluation tools for prognosis from good recovery to death; Unable to distinguish VS from MCS
CRS-R	Auditory, visual, motor, oral, communication, arousal	25	5 (23)	The specific behavioral response to specific sensory stimuli; Each item is standardized and operable; It is a relatively recognized evaluation scale
FOUR	Eye response, motor response, respiration, brainstem reflexes	10	4 (15)	Make up for the defect that the language function in GCS scale can’t be tested due to mechanical ventilation; Have the value of outcome prediction
WNSSP	Visual tactile, olfactory, arousal/attention, auditory, expressive communication	45	5 (32)	Needs to rely on visual understanding and tracking
WHIM	Basic behaviours, social/ communication, attention/ cognitive, orientation/memory	30 – 60	4 (58)	For detecting the changes of consciousness recovery in each stage; It takes longer to evaluate
SMART	Auditory, vision, tactile, olfactory, gustatory, wakefulness, motor, communication	60^+^	8 (60)	Evaluation from multi-modal sensory stimulation response; It takes a long time; It requires higher professionalism
DOCS	Auditory, visual, tactile, sensory, swallowing, olfactory	45	8 (23)	Evaluate the overall level of neurobehavioral response
DRS	Awakening and awareness, cognitive dependence, social and psychological adaptability	10	8 (32)	Quantitative evaluation of disability degree of patients with severe brain injury in the process from coma to return to society
CNC	Visual, auditory, command following, threat response, olfactory, tactile, pain, vocalisation	10	8 (32)	Rappaport is an extension of its previous DRS scale, which is easy to learn, fast to complete, effective and predictive of prognosis

GCS, Glasgow coma scale; GOS, Glasgow outcome scale; CRS-R, coma recovery scale-revisited; FOUR, full outline of unresponsiveness score; WNSSP, western neurosensory stimulation profile; WHIM, Wessex head injury matrix; SMART, sensory modality assessment technique; DOCS, disorders of consciousness scale; DRS, disability rating scale; CNC, coma/near-coma scale

Among these measures, the CRS-R uniquely incorporates diagnostic criteria for coma, VS, and MCS into its administration and scoring system. It has been particularly effective for assessing consciousness changes before and after treatment. Schiff et al. [6] utilized the CRS-R to measure improvements in patient awareness following DBS. Similarly, Magrassi et al. [25], Dang et al. [31], and Adams et al. [66] reported changes in CRS-R scores after DBS. While these scales provide a foundation for quantifying DBS-induced conscious behavior changes, further investigation into the neural regulatory mechanisms of DBS is necessary.

### Neuroelectrophysiology

#### EEG

EEG is of great value in consciousness research, offering precise measurement of neural electrical activity with high temporal resolution [67, 68] (Fig. 1f). Numerous EEG-based features have been put forward to elucidate the pathophysiology of DOC, and various clinical assessment methods have also been described [69]. Since basic EEG features are correlated with synaptic activity and subcortical influences within cortical circuits over time, clinical EEG traces provide insights into the functional integrity of thalamocortical networks in DOC patients [70, 71]. Based on the current understanding of brain oscillations, some EEG patterns in DOC may indicate disconnections (or deafferentation) within and between cortical and subcortical structures [72–74].

The mesocircuit hypothesis suggests that severe brain injuries, regardless of the cause, may directly (thalamocortical and brainstem-thalamus) or indirectly (striatum-thalamus) reduce the activity of central thalamic neurons within the ascending arousal system [75]. Consequently, there is a hierarchical relationship between the extent of deafferentation, thalamic activity patterns, and EEG patterns. CT neurons can be classified based on the levels of deafferentation: quiescent neurons (“A type” and “B type”), neurons entering a bursting mode (“C type”), and neurons transitioning into a tonic firing mode (“D type”) [76]. These modes are believed to signify an increasing excitatory drive, particularly within anterior forebrain thalamocortical circuits [41]. Restoration by the thalamus and a shift to tonic firing support typical wakeful EEG activity, characterized by specific oscillations (α and β), corresponding to the D-type pattern [37].

Forgacs et al. [77] evaluated DOC patients using MRI and fluorodeoxyglucose positron emission tomography (FDG-PET), correlating the findings with EEG results. They reported intact wakeful EEG structures, including dominant posterior rhythms, across all patients. A new EEG classification based on three descriptors—predominant background frequency, anterior–posterior gradient organization, and the presence of diffuse/focal slowing —has been proposed. This classification encompasses 4 EEG categories: normal, mild abnormal, moderate abnormal, and severe abnormal [77]. It demonstrated 61% sensitivity and 75% specificity for diagnosing MCS in DOC when a normal/mildly abnormal background was present. Estraneo et al. [78] introduced a fifth category, the low voltage pattern, which is more commonly observed in VS/UWS patients. These two studies are strongly recommended in the latest European Academy of Neurology DOC Diagnostic Guidelines for DOC diagnosis [79]. Analysis indicates that a normal or mildly abnormal background reliably identifies MCS with high specificity but low sensitivity.

Several studies have explored clinical EEG features for predicting the prognosis of DOC patients. Bagnato et al. [80] initially demonstrated the prognostic utility of EEG for 3-month outcomes using the Synek classification. Subsequent studies focused on a limited set of standard EEG features—total amplitude, dominant frequency, and reactivity [collectively termed the Amplitude Frequency Reactivity (AFR) score]—which were correlated with 3-month DOC outcomes [80, 81]. Specifically, a reduced overall EEG amplitude and a dominant δ frequency (< 4 Hz) were significantly associated with poorer outcomes, while a dominant α frequency and preserved reactivity predicted recovery [81].

Functional connectivity (FC) techniques, particularly those based on EEG, hold significant potential by assessing cortical integrity and inter-regional communication. EEG-based functional connectivity (EEG-FC) evaluates the synchronization and interactions between neural activities across brain regions, providing a non-invasive, cost-effective, and routine approach for assessing brain function in DOC [82]. However, caution is necessary: improved FC after neuromodulatory interventions, such as transcranial direct current stimulation (tDCS) or pharmacological treatments, does not always equate to complete consciousness restoration, as increased connectivity may indicate partial recovery or compensatory mechanisms rather than full functional restoration [83, 84]. Additionally, patients with similar clinical diagnoses may exhibit distinct FC patterns, complicating the interpretation of results. Furthermore, the effects of neuromodulatory interventions on EEG-FC can be transient and may not consistently correspond with long-term clinical improvement. Therefore, while EEG-FC shows promise as a tool for assessing consciousness in DOC and may emerge as a new standard for evaluating cortical integrity due to its correlation with clinical measures like the CRS-R, increased FC should be interpreted with caution, as it may not signify complete consciousness recovery [84].

#### Quantitative EEG (QEEG)

QEEG analysis can assess the integrity and activation level of the thalamic-cortical system by examining the EEG signal power spectrum. Current research reveals a significant decrease in θ (4—8 Hz) and α (8—13 Hz) frequency bands in VS/UWS patients in comparison with MCS patients, while the δ (< 4 Hz) frequency band shows the opposite trend [85–87]. QEEG studies support a negative correlation between EEG spectral slowing and CRS-R scores, validating the clinical concept of EEG slowing. Sitt et al. [88] analyzed 92 EEG-derived metrics in 181 DOC patients, finding that low-frequency energy, EEG complexity, and information exchange serve as crucial indicators for distinguishing levels of consciousness, effectively differentiating MCS from VS/UWS.

Various EEG analysis methods have been developed for processing brain signals, including entropy, which quantifies regularity. Higher entropy values signify a state closer to wakefulness, while lower values imply a state closer to unconsciousness. This metric facilitates the exploration of the relationship between brainwave frequency fluctuations and consciousness levels [69, 89]. Several computational approaches calculate entropy, such as approximate entropy, Lempel–Ziv complexity, and cross-entropy, which may identify VS/UWS and MCS or correlate with clinical scores [90]. The percentage of α microstates within the combination index (power in α and δ frequency bands, entropy, and microstates) has been proven effective in distinguishing VS/UWS from MCS [91].

In recent years, the field of DOC has adopted machine learning (ML) techniques, such as decoding and multivariate pattern analysis, for analyzing EEG data. These methods involve training classifiers on EEG data and diagnostic labels, enabling them to learn patterns that distinguish consciousness states. The reliability of these classifiers is evaluated on independent datasets to determine their diagnostic accuracy. The ultimate aim is to develop an automated, standardized bedside assessment tool for DOC [92]. Meanwhile, there are non-invasive EEG-based brain-computer interfaces (BCI) being utilized in the diagnosis and prognosis of patients with DOC. By analyzing the EEG signals, medical professionals can obtain insights into the neural activity and potential consciousness levels of patients with DOC [93, 94].

#### Event-related potential (ERP)

ERP is a valuable tool for assessing sensory information processing and basic cognitive functions in DOC or comatose patients [95–98]. A meta-analysis has shown that late evoked potentials, particularly P300 waves, are potent predictors of consciousness recovery in DOC patients [99]. ERP, or “endogenous response”, reflects neural activity and cognitive processing during embedded stimulus tasks. Using multiple ERP markers in a single test improves diagnostic sensitivity, facilitating the identification of patient-specific residual cognitive functions. Recent ERP research has focused on auditory processing levels (auditory, perceptual, and semantic) [100, 101] and various cognitive dimensions, such as novel spatial attention markers [102]. For instance, Gui et al. [101] recently examined rhythmic brainwave responses associated with tracking words, phrases, and sentences using a hierarchical language paradigm. Their findings in predicting the prognosis of DOC patients have been promising, achieving 80% accuracy.

#### Transcranial magnetic stimulation (TMS)-EEG

A method for directly assessing functional integration and differentiation within the thalamocortical circuit involves measuring the complexity of brain response to stimulation. The degree to which different neuron groups interact as an integrated whole (integration) to generate complex dynamics (differentiation) can be evaluated by applying transcranial magnetic stimulation and recording EEG responses [103]. This approach enables direct cortical neuron stimulation and measures the impact of initial activation on the brain with high spatiotemporal resolution.

Two studies on small groups of DOC patients revealed a similar correlation between TMS-evoked potentials (TEP) and consciousness levels [104, 105]. Casali et al. [106] subsequently developed the Perturbational Complexity Index (PCI), which quantifies the information generated by large-scale causal interactions within the thalamocortical system and presents it as a single score. A recent study used TEP data from a large DOC cohort (38 MCS and 43 VS/UWS patients) to calculate the PCI, demonstrating very high sensitivity (94%) in detecting MCS [107]. This sensitivity may be attributed to the inherent causal mechanism indicated by cortical perturbations, increasing δ power/synchrony [72], reduced long-range connectivity [83], and the loss of complexity often observed in unconscious patients [88, 107]. These electrophysiological studies enhance our comprehension of the effects of DBS in DOC patients, underscoring the necessity for further research to elucidate the treatment mechanisms of DBS.

### Neuroimaging

Neuroimaging is essential for exploring the neural basis of human consciousness (Fig. 1g). Structural imaging identifies and localizes brain damage in DOC patients, while functional imaging reveals the activity patterns and mechanisms underlying this damage. Traditional head computed tomography is accessible, facilitating rapid data collection, lesion localization, and early mortality prediction for acute DOC cases, including TBI, hemorrhage, and hypoxic-ischemic encephalopathy. However, due to its low sensitivity, computed tomography is rarely used alone in clinical practice to detect most physical abnormalities in DOC patients [79]. Structural MRI is the most direct approach for visualizing brain abnormalities and diagnosing DOC. Since DOC patients often present with diverse cortical injuries, an injury network mapping technique has been proposed to study distributed cortical networks related to arousal, enabling automatic organization segmentation for volume or morphological analysis. A recently applied enhanced tree technique in regional volume information analysis achieved 90–98% classification accuracy for VS/UWS and MCS [108, 109].

#### Diffusion tensor imaging (DTI)

DTI is a specialized form of MRI. With the use of DTI, a strong correlation was observed between widespread white matter disconnection, particularly in the fornix, and DOC following severe brain injury [110]. Abnormal structural connections have also been identified within the basal ganglia, frontal cortex, and thalamus in DOC patients [111]. The degree of interhemispheric disconnection, such as complete separation, serves as an independent biomarker for consciousness [112]. An innovative DTI study found that ML algorithms achieved up to 100% accuracy in distinguishing thalamocortical tracts reaching the frontal lobe, parietal lobe, and somatosensory motor area [113]. Reduced connectivity in subcortical arousal pathways has recently been shown to be a sensitive indicator for DOC [114]. Recent multicenter studies demonstrated the predictive value of DTI for 1-year outcomes, surpassing structural and clinical assessments for both traumatic and anoxic patients [115, 116]. Additionally, the combination of DTI with MRI spectroscopy predicted long-term outcomes for traumatic patients, achieving 86% sensitivity and 97% specificity for non-recovery prediction after 1 year [117]. In diagnostic applications for DOC patients, a recent study employed DTI to identify measures potentially distinguishing VS/UWS from MCS patients. Diffusion patterns in MCS and VS/UWS patients differed significantly in subcortical white matter and thalamic regions, but not in the brainstem. Furthermore, DTI characterized etiological differences in VS/UWS patients, with brainstem abnormalities confined to the traumatic brain-injured group [118].

#### Functional MRI (fMRI)

fMRI technology assesses subtle changes in magnetic resonance signals resulting from blood oxygen level-dependent (BOLD) variations within microvasculature across brain regions. The evaluation of time-related BOLD signals with fMRI is beneficial for evaluating DOC patients.

Among DOC patients, beyond the limited bedside assessments, communication ability can be assessed through advanced techniques (e.g., fMRI, EEG, or ERP). Notably, PET and fMRI reveal brain metabolic activity, uncovering residual higher-order brain functions in patients otherwise diagnosed with VS/UWS, including cross-sensory processing, language, learning, emotion, and pain perception [47, 119, 120]. The preserved connectivity within isolated networks provides evidence of severe impairment in expressing the functions of the surviving brain modules, which is interpreted as an indication of residual covert cognition or consciousness. Observing the brain activation areas reflecting consciousness and cognition in only a few subjects through neuroimaging highlights the ability to generate voluntary “brain behaviors”, suggesting partially preserved consciousness [121].

A preliminary study indicated that connectivity configuration occurred at a lower frequency in VS/UWS patients than in controls [122]. A multicenter study confirmed that consciousness relies on the brain’s ability to sustain rich and dynamic activity [123]. Subsequent research demonstrated that integrating dynamic functional connectivity in predictive modeling strategies can forecast CRS-R scores and reduce individual heterogeneity in statistical analyses [124]. Schiff et al. [9] employed fMRI in a cross-control experimental paradigm, underscoring the importance of preserving brain function in DOC patients to achieve the positive regulatory effects of DBS. Additionally, Raguz et al. [29, 125] observed that DBS could lead to volume growth in the cerebral cortex and subcortical structures through brain MRI scans, suggesting this mechanism may contribute to enhanced patient awareness via DBS.

#### PET-computed tomography (PET-CT)

PET-based diagnosis of metabolic abnormalities in the brain has driven the advancements in neuroimaging. Early functional imaging patterns using FDG and oxygen-15 labeled water (H₂^15^O) are commonly utilized PET molecular markers for the evaluation of DOC. FDG-PET studies have shown a 40 – 50% decrease in whole-brain metabolism in DOC patients [119] and reduced cortical effective connectivity in VS/UWS patients [126]. H₂^15^O-PET studies suggest that consciousness recovery seems to be associated with restored functional connections between the thalamus and cortex [127, 128]. A comparative study on PET activation across DOC states revealed that the metabolism of the frontoparietal network was better preserved in MCS patients compared to controls [129]. One study even proposed that FDG-PET could predict long-term recovery in VS/UWS patients, showing higher predictive accuracy than subsequent fMRI evaluations [130].

Through reciprocal interactions with subcortical neurons, matrix neurons support wakefulness and set perceptual thresholds, while core neuron cortical interactions maintain content and enable perceptual consistency [131]. Annen et al. [132] used FDG-PET and EEG to evaluate cortical and subcortical regions in healthy individuals and DOC patients. Their findings highlight a close correlation between subcortical metabolic balance and consciousness, with increased metabolism being associated with higher consciousness levels. Notably, the relationship between parietal-occipital and lateral frontal cortices and subcortical glucose uptake is significant in DOC patients, forming the frontoparietal network (FPN) critical for identifying consciousness. Linear mixed-effects analysis disclosed a negative correlation between θ-wave power and glucose uptake in DOC, while α-wave power positively correlated with glucose uptake in both DOC and recovered patients [133].

Behavioral, electrophysiological, and neuroimaging studies provide compelling evidence of the regulatory impact of DBS on DOC. In their review of DBS research within DOC, Vanhoecke et al. [134] and Rezaei Haddad et al. [135] emphasized the importance of neuroimaging and neuroelectrophysiological techniques in evaluating the regulatory effects of DBS.

## Technical issues concerning DBS treatment of DOC

### Central thalamic nucleation localization and segmentation

The thalamus, a critical component of the diencephalon, is indispensable for various neurological processes [136–140]. Despite its broad division into anterior, lateral, and medial subnuclei, distinguishing these units in clinical MRI scans remains challenging. Nevertheless, identifying individual thalamic subnuclei is vital for understanding thalamic anatomy and function and for guiding interventions such as DBS (Fig. 1h). Thalamic mapping techniques over the past two decades can be classified into two main categories: histology-based and MRI-based atlases. While histology-based atlases remain the gold standard, MRI-based atlases offer superior speed and capacity. The brain parcellation criteria in both approaches focus on substructures that are structurally or functionally similar [139].

Histological atlases enable detailed delineation of thalamic regions. The atlases developed by Krauth et al. [140] and Gallay et al. [141] segment the unilateral thalamus into 7 subregions using chemical markers. Stereotactic atlases divide the unilateral thalamus into 38 subregions, and Goto et al. [142] created a 3D atlas with 107 subregions. A recent probabilistic atlas combining histological and in vivo structural MRI data produced 13 parcellated subregions [143]. However, these methods are invasive, labor-intensive, and require manual labeling.

MRI-based atlases offer non-invasive and efficient alternatives to histological atlases, including those derived from structural MRI, fMRI, and diffusion MRI. Middlebrooks et al. [144] enhanced the fast gray matter acquisition T1 inversion recovery MRI sequence for improved visualization, while Traynor et al. [145] introduced a T1-T2 MRI method with 6 thalamic subregions. Techniques such as magnetization-prepared rapid gradient-echo MRI provide distinct thalamic nuclei contrast [146], and Su et al. [147] proposed a multi-atlas segmentation optimized for the thalamus, yielding 12 subregions. Susceptibility-weighted imaging and quantitative susceptibility mapping facilitate structure-based thalamic parcellation [148, 149]. MRI-based atlases are beneficial for non-invasive and efficient thalamic research.

fMRI-based atlases extract BOLD signals from resting-state fMRI data, facilitating the assignment of voxel to subregions. Zhang et al. [150] identified 5 and 31 functional thalamic subregions, respectively, through different methods. Kumar et al. [151] applied transient BOLD signal correlation to enhance histological consistency, resulting in 15 subregions. Tian et al. [152] introduced an innovative method using eigenvectors and eigenvalues from a group-level similarity matrix to perform “fiber tracking”, dividing the thalamus into 8 regions.

Diffusion MRI-based atlases provide valuable insights into the anatomical connections and microstructures of thalamic nuclei. Patriat et al. [153] identified 9 cortical regions, offering strong functional confidence but presenting weaker structural consistency. Yang et al. [154] employed DTI to divide the thalamus into 16 subregions. Jonasson et al. [155] improved this approach through the level set algorithm, while Ye et al. [156] proposed a more flexible method based on the mean shift algorithm to overcome existing limitations.

The advancements in the spatial resolution of diffusion MRI have facilitated the estimation of fiber orientation distribution. Kumar et al. [157] employed the diffusion tensor model and high-resolution diffusion MRI for voxel clustering based on dominant diffusion orientation and spatial location. Although the tensor model is simple, the orientation distribution function (ODF) offers superior parcellation performance in high angular resolution diffusion imaging. Battistella et al. [158] classified the thalamus into 7 regions using ODF data, aligning with Morel’s histological atlas. The eighth-order spherical deconvolution for ODF estimation demonstrated remarkable regional specificity, with distinct differences between thalamic regions [159]. These techniques provide valuable tools for thalamic research, each with its unique advantages and challenges, enhancing our understanding of thalamic organization and function. Further progress in thalamic parcellation will deepen our knowledge of brain connectivity and function in health and disease.

In summary, this overview of thalamic parcellation methods and individualized atlases categorizes them into four groups: histology-based, structural MRI-based, fMRI-based, and diffusion MRI-based. Challenges remain, such as the absence of a universally accepted benchmark, limited integration of multimodal information, and restricted options for subcortical nuclei mapping. While resting-state fMRI is commonly used for individualized subcortical partitioning, diffusion MRI – which offers stable fiber orientations and structural connectivity – is still underutilized for atlas individualization. Addressing these challenges will advance our understanding of the thalamus and its role in brain function.

### Microelectrode signal characteristics in the CT

According to the mesocircuit hypothesis, the CT plays an essential role in the mechanisms underlying DOC [10, 23, 26, 160]. The CT is also a primary target for DBS in DOC and anesthesia recovery research [9, 25, 34, 161]. Hence, current research on microelectrode signal of DOC primarily focuses on the CT [161, 162], facilitating target localization, understanding electrophysiological mechanisms, and guiding individualized parameter settings of CT-DBS.

Given that trauma-induced changes and brain atrophy can affect the accuracy of map localization, microelectrode data recording from the CT is frequently conducted before DBS electrode implantation to confirm the optimal placement (Fig. 1i) [9, 25, 154, 155]. Since CT activity is already reduced with impaired consciousness, the impact of intraoperative anesthesia during microelectrode recording must be minimized [23]. Magrassi et al. [25] decreased the end-tidal concentration of sevoflurane to 0.5 – 0.7%, which was well below the MAC90 threshold, to maintain signal quality.

Microelectrode signals frequently undergo single-neuron activity analysis for the characterization of the CT. Once the activity of individual neurons is identified through spike detection and classification, features such as action potential amplitudes, firing rates, spike intervals, and burst firing patterns are extracted [163]. These single-neuron activity features assist in distinguishing thalamic nuclei, localizing CT-DBS targets, and evaluating CT activity at different consciousness levels [164, 165]. For instance, Redinbaugh et al. [55] observed significantly lower firing rates of CT neurons during anesthesia or non-rapid eye movement (NREM) sleep, which were associated with lower consciousness levels, compared to the waking state. Magrassi et al. [162] classified CT neurons into spiking neurons (SN), which fire single action potentials, and bursting neurons (BN), which fire action potential trains of varying durations. They found a 50% decrease in active neurons in the CT of VS/UWS patients compared to MCS patients, with less than 17% of neurons exhibiting both types of bursting. These findings suggest that lower consciousness levels correspond to reduced CT firing rates, indicating disrupted and desensitized CT activity in DOC patients. However, consciousness assessment remains limited to a coarse-grained level due to the small sample size.

Drover et al. [76] proposed an “ABCD” classification scheme that organizes the relationship between deafferentation levels in DOC following severe brain injuries, CT state, and expected EEG patterns. The ABCD model categorizes degrees of cerebral deafferentation and their impact on neocortical neurons, as measured through EEG. A key prediction is that graded functional deafferentation across the neocortex correlates with an anterior–posterior gradient, resulting in progressive patterns of neuronal firing rates in the EEG power spectrum, aligned with cortical metabolic activity from severe downregulation to full recovery. Patients with severe anoxic brain injuries in a vegetative state may exhibit these “A-type” EEG spectral features [57]. The A-type spectral background is characterized by slow EEG oscillations of approximately 1 Hz, indicating complete functional cortical-thalamic dedifferentiation. In contrast, the oscillatory frequency of the type B cortical circuit ranges between 5 and 9 Hz, reflecting a degree of cortical activation but still a relatively low dedifferentiation level, leading to a quiescent neuronal state. In a healthy and intact cerebral cortex without deafferentation, the resting average membrane potential is −55 mV, and EEG power spectra show a peak in the α range (8–12 Hz) with additional peaks at higher frequencies (“D-type” spectra) [10]. For patients recovering from DOC, the full restoration of thalamic facilitation and a shift to ubiquitous tonic firing restore typical wakeful EEG activity, characterized by α and β oscillations, by the “D-type” pattern.

The mesocircuit hypothesis delineates the ABCD model, reflecting the changes in CT activity across consciousness levels [10, 160, 166]. Giacino et al. [161] observed minimal CT activity in three MCS patients, correlating with the lowest consciousness level (AB phase) of the ABCD model. As consciousness levels increase towards MCS, representing the C phase, the CT often exhibits bursting activity. Based on thalamocortical dysrhythmia, Llinás et al. [167, 168] proposed that CT neurons begin low-frequency bursting when they reach the depolarized membrane potential. This bursting activity has been associated with positive neurological symptoms, including neurogenic pain, abnormal movements, tinnitus, epilepsy, and neuropsychiatric symptoms [169]. Furthermore, CT burst activity is higher under anesthesia and NREM sleep than in wakefulness [55]. CT activity normalizes as consciousness approaches full restoration, moving to the D phase, characterized by tonic discharges. However, direct evidence for CT activity in phases C and D is lacking, necessitating future large-scale validation.

In DOC patients, less damage is correlated with better-preserved CT discharge activity. Enhanced thalamic activity may aid in restoring the thalamocortical region, the ascending arousal network, and potentially the entire functional connectivity of the brain, thereby improving prognosis [35, 67, 170–174]. The potential of CT microelectrode signals in predicting consciousness recovery presents a promising avenue for future research.

### LFP signaling in the CT

LFP represents the low-frequency portion (below approximately 300 Hz) of extracellular field potentials generated by membrane currents in neurons near the recording electrode (within 0.5–3 mm of the electrode tip) [175]. Distinct LFP rhythms or connectivity changes across brain regions may signify specific neuronal activity states or disease-related functions. In consciousness research, LFP recording has been pivotal for examining rhythmic properties and connectivity features during sleep, anesthesia, and consciousness disturbances. This research involves correlating consciousness shifts across brain regions by analyzing LFP cadence and connectivity characteristics (Fig. 1j).

Consciousness-related LFP studies have primarily focused on rhythmic components, including δ, θ, α, β, and γ rhythms. Donoghue et al. [176] recorded LFP in the hypothalamus of anesthetized rhesus monkeys, identifying 1 Hz slow-wave oscillations during unconscious states. Similarly, Xu et al. [177] observed this LFP pattern in rodent models of post-epileptic coma induced by thalamic CL-DBS. Both studies demonstrated arousal and electrophysiological reversals in unconscious states following bilateral CT electrical stimulation, accompanied by improved behavioral responses. Huang et al. [178] monitored thalamic function in DOC patients using LFP recorded by a macroelectrode, finding higher amplitude 10 Hz oscillations in the CT of MCS patients compared to VS/UWS patients, suggesting a possible connection to residual consciousness. Wojtecki et al. [24] induced LFP activity in the CT of a DOC patient using familiar language stimulation, resulting in modulation of oscillatory activity in the θ and β bands within the central thalamus and coupling an increase in thalamocortical coherence in the θ band. He et al. [179] found that, following DBS treatment in 23 DOC patients, 11 (47.8%) showed consciousness improvement. By recording CM-Pf nucleus LFP, a positive correlation was noted between higher firing rates, increased multi-unit activity (MUA) raw power in the high γ band, enhanced normalized α band power, and stable MUA in the θ band, correlating with better outcomes.

Cortical LFP activity also varies with consciousness levels. In 2018, Nourski et al. [180] performed electrocorticography (ECoG) on the auditory and prefrontal cortex during an auditory task under propofol anesthesia. They identified local and global effects as mean evoked potentials, detecting high γ frequency bands. During wakefulness, both effects were observed in certain cortical regions, with mean evoked potentials being more widespread than high γ activity. A study conducted by Nourski et al. [181] in 2021 recorded ECoG signals in the primary auditory, auditory-related, and prefrontal cortices of epilepsy patients during an auditory vowel sequence task in both awake and anesthetized states. They found that as anesthesia deepened, auditory evoked potentials (AEPs) diminished in the superior temporal gyrus and adjacent auditory cortex. The core auditory cortex exhibited reduced AEP amplitude, changed high γ band activity, and increased inter-trial response variability.

Functional connectivity between thalamocortical and cortical regions is crucial for consciousness. Donoghue et al. [176] recorded LFP signals in macaques under propofol anesthesia, noting reduced coherence in higher frequency bands during unconsciousness. In 2020, Redinbaugh et al. [55] observed increased δ band coherence but decreased α and low γ coherence in anesthetized macaques, indicating altered cortical processing. Thalamocortical coherence decreased with anesthesia but improved with effective stimulation. Additionally, Wojtecki et al. [24] revealed enhanced thalamocortical θ coherence in DOC patients in response to language stimuli.

In awake and anesthetized primates, DBS targeting the intralaminar thalamus has been demonstrated to reverse the unconscious state induced by propofol. Synchronized EEG and fMRI revealed that electrical stimulation enhanced the identification and output of the intralaminar thalamus drive networks responsible for rapid arousal during slow-wave oscillations [182]. Consciousness was linked to the functional connectivity of thalamic, thalamocortical, and cortical pathways, such as the ascending reticular activating system, central circuit, and cortical network, which is weakened in DOC patients. Solovey et al. [183] showed increasing instability and greater coherence across cortical regions as consciousness returned after anesthesia. Propofol anesthesia induced persistent slow oscillations in visual and oculomotor cortical networks [184], while slow cortical potentials persisted in the somatomotor cortex during anesthesia induction [185]. Matthew et al. [186] demonstrated a shift in functional connectivity from the temporal to frontal cortex during reduced consciousness, resembling changes observed under anesthesia and sleep.

Currently, there is no specific animal model for DOC research, which primarily focuses on altered consciousness states during sleep, anesthesia, and seizures. Advances in neurophysiological techniques can deepen our understanding of individual mechanisms in consciousness disorders, improving clinical diagnosis, treatment, and prognosis prediction [187].

### Computational modeling of therapeutic DBS for DOC

Computational modeling serves as an indispensable tool for comprehending the pathogenesis of DOC and analyzing the oscillatory dynamics among arousal, awareness, and movement states in DOC patients. This goal-driven state modeling of DOC bridges the gap between the intrinsic properties of the brain and external behaviors, offering critical insights for interpreting neuromodulation mechanisms and developing neuromodulation strategies (Fig. 1k).

Multi-level modeling of microscopic neurons, mesoscopic neural clusters and circuits, and macroscopic brain states enable a virtual reconstruction of DOC. At the microscopic level, computational models depict the dynamics of cell membrane activity, changes in ion concentration, and characteristics of ion channels, analyzing the nonlinear mechanisms of neuron subtypes and dendrites [46, 188, 189]. At the mesoscopic level, these models simulate essential neural circuits, analyzing the looping mechanisms of DOC states, neural regulation, and arousal [45, 190, 191]. At the macroscopic level, the model employs multi-dimensional data (e.g., MRI, EEG, and PET signals) to reconstruct the three-dimensional (3D) brain state, reproducing the “personality state” of DOC [45, 192].

The interaction of neural mechanisms over both short and long timescales is of crucial significance for consciousness [193]. Annemarie et al. [194] proposed that the brain’s intrinsic temporal hierarchy, measured by the autocorrelation window (ACW) and intrinsic neural timescales (INT), is associated with cognitive functions. In primates, a longer prefrontal ACW is correlated with longer task delays. Human fMRI and EEG studies have demonstrated a direct correlation between the resting-state ACW and higher-order cognition. Buccellato et al. [193] compared EEG measures across consciousness levels, finding a significant correlation between ACW and alpha peak frequency (APF) in conscious states, which was disrupted in unconscious states. This supports the alignment of neural timescales with environmental inputs as being essential for consciousness. Zilio et al. [195] studied EEG dynamics in locked-in syndrome (CLIS) patients and healthy individuals under anesthesia and sleep states, unveiling that EEG temporal features, such as power-law exponent (PLE) and Lempel–Ziv complexity (LZC), distinguished arousal levels in CLIS patients, indicating reduced and unstable consciousness. This intra-individual variability may function as a biomarker for arousal/vigilance fluctuations.

From a dynamic perspective, state-space models exhibit the evolution of neural states under stimuli. Microscopic system state-space models have shown that alterations in the gating variables of T-type calcium ion channels in DOC can induce changes in the firing of thalamic neurons, leading to burst firing of thalamic cells and approximately 3–7 Hz synchronic oscillatory behavior in the thalamic nucleus [46, 196].

Additionally, multi-compartmental cortical neuron models suggest that DOC may stem from weakened inter-compartmental coupling in cortical neurons, hindering the projection of conscious content to the thalamic nucleus through specific coding pathways. Schiff et al. [9, 35] further put forward the mesocircuit model at the network level, elucidating DOC mechanisms and providing a framework for dynamic explanations and investigations of stimulation mechanisms at diverse arousal, awareness, and motor function levels. Redinbaugh et al. [55] found that 50 Hz stimulation in the CT of primates could evoke awakening in anesthetized subjects, observing a rhythm transition in thalamic and cortical networks from δ (1–4 Hz) to α (8–13 Hz). This research links microscopic neural dynamics to macroscopic behaviors, offering an academic interpretation of neural properties and network rhythm transitions from anesthesia to arousal.

The rapid growth of neural data resulting from advancements in electrophysiology and imaging techniques poses challenges to traditional neuroscience in analyzing the perturbation mechanisms of consciousness. The incapacity to effectively analyze this data has impeded the further development of arousal techniques for consciousness disorders. However, artificial intelligence (AI), particularly deep learning, offers novel solutions.

Deep learning can reduce data dimensionality, simplify the representation of brain dynamics, decode hidden features of consciousness disturbances, and facilitate the study of neural regulation to arouse consciousness [197]. Virtual brain models based on structural connectivity estimation, functional connectivity mapping, and spatiotemporal dynamics modeling of individual nodes can characterize the features of a whole-brain network. A mean-field model, describing brain dynamics at a single point in time and space, can link networks of coupled oscillators on a macroscopic scale [49, 198]. This explicit depiction of macroscopic dynamics in microscopic terms offers a pathway to uncover the relationship between network coupling properties and neuronal oscillator dynamics in consciousness disorders. Consequently, consciousness perturbation state models act as effective tools for analyzing the dynamic properties of these systems, facilitating identify and regulate network states at different consciousness levels.

## Unmet needs and future perspectives in DBS for DOC

Despite significant advancements in treating DOC through DBS, numerous unmet needs remain, presenting abundant opportunities for improvement and discovery. One of the most urgent challenges is accurately identifying patients who will benefit the most from the intervention. Currently, neuroimaging and electrophysiological detection technologies provide valuable insights from various perspectives, facilitating patient stratification and personalized treatment evaluation. However, clinical adoption has been slow, hindered by a shortage of high-quality research to support the inclusion of these detection methods in clinical guidelines.

Secondly, precise target localization and implantation are prerequisites for effective treatment. Traditionally, the identification of thalamic nuclei has relied on histological atlases. Recently, MRI-based atlases have proved superior, offering a rapid, non-invasive, and accurate alternative. These techniques have deepened our comprehension of thalamic regions associated with consciousness, enabling further exploration of thalamic organization and function.

Currently, DBS practices frequently rely on a trial-and-error approach to determine optimal stimulation settings, restricting precision and efficiency. Nevertheless, goal-driven modeling of DOC states presents a ground-breaking approach, enabling a comprehensive, multi-scale examination of DOC brain states, ranging from microscopic neuronal to macroscopic observational levels. This approach facilitates the simulation of brain state changes during stimulation, paving the way for adaptive closed-loop neuromodulation strategies. Consequently, DBS stimulation could be tailored for shorter durations and greater efficacy in restoring consciousness.

The next phase should entail rigorous large-scale controlled studies, with an emphasis on leveraging DBS to induce neuroplasticity within the brain to elicit sustained, self-perpetuating recovery mechanisms for DOC patients. The integration of AI and AI algorithms for analyzing complex neurological data holds the potential to revolutionize patient assessment, treatment planning, and DBS outcome prediction. This might lead to the development of more personalized, adaptive, and effective DBS therapies, transforming DOC treatment.

## Conclusions

In conclusion, DBS for treating DOC holds considerable promise in neurology and neuroscience. This review explored the complex details of DBS, examining its anatomical and functional bases within the CT and various technological advancements and methodologies aimed at optimizing its application. The significance of DBS in treating DOC lies in its potential to restore or enhance consciousness in patients with brain injuries or disorders. The CT, essential for regulating cognitive and emotional functions, is a critical target for treatment. DBS offers hope to patients and their families by potentially improving the quality of life and facilitating functional recovery. However, DBS in treating DOC has limitations. The variable outcomes among patients, the complex mechanisms of DBS therapy, and the long-term effects and potential side effects of DBS remain ongoing challenges. Addressing these constraints will require further rigorous research and development.

The prospects for DBS in the treatment of DOC are promising. Researchers will continue to refine DBS techniques, enhancing the precision of electrode placement and stimulation parameters. A deeper comprehension of the biological mechanisms underlying DOC and the intricate neural networks involved holds the potential for more personalized and effective treatments. With advancements in neuroscience and medical technology, innovative therapeutic approaches may emerge, providing even greater hope for individuals with DOC.

In summary, DBS therapy represents a significant advancement in the treatment of DOC, holding the potential to improve outcomes and enhance the quality of life for affected individuals. While challenges and uncertainties persist, ongoing research and technological progress are essential to fully unlocking DBS’s potential as a transformative treatment for DOC in the future.

## Data Availability

Not applicable.

## Abbreviations

ACRM: American congress of rehabilitation medicine
ACW: Autocorrelation window
AEP: Auditory evoked potentials
AFR: Amplitude frequency reactivity
AI: Artificial intelligence
APF: Alpha peak frequency
BCI: Brain-computer interfaces
BN: Bursting neurons
BOLD: Blood oxygen level-dependent
CL: Central lateral
CLIS: Locked-in syndrome
CM-Pf: Center median parafascicular complex
CNC: Coma/near coma scale
CRS-R: The coma recovery scale-revised score
CT: Central thalamus
CT-DBS: Central thalamus-deep brain stimulation
DBS: Deep brain stimulation
DMN: Default mode network
DOC: Disorders of consciousness
DOCS: Disorders of consciousness scale
DRS: Disability rating scale
DTI: Diffusion tensor imaging
ECN: Executive control network
ECoG: Electrocorticography
EEG: Electroencephalogram
EEG: Electroencephalography
EEG-FC: EEG-based functional connectivity
ERP: Event-related potentials
FC: Functional connectivity
FDG: ^18^F-fluorodeoxyglucose
FDG-PET: Fluorodeoxyglucose positron emission tomography
fMRI: Functional magnetic resonance imaging
FOUR: Full Outline of UnResponsiveness scale
GCS: Glasgow coma scale
GOS: Glasgow outcome scale
GPi: Globus pallidus
GWTs: Global workspace theories
HOTs: Higher-order theories
IIT: Integrated information theory
INT: Intrinsic neural timescales
LFP: Local field potential
LZC: Lempel-Ziv complexity
MAC: Minimum alveolar concentration
MCS: Minimally conscious state
ML: Machine learning
MRI: Magnetic resonance imaging
MSNs: Medium spiny neurons
MUA: Multi-unit activity
NREM: Non-rapid eye movement
ODF: Orientation distribution function
PCI: Perturbational complexity index
PLE: Power-law exponent
QEEG: Quantitative electroencephalography
RDS: Rappaport disability rating scale
SECONDs: Simplified evaluation of consciousness disorders
SMART: Sensory modality assessment technique
SN: Spiking neurons
TBI: Traumatic brain injury
tDCS: Transcranial direct current stimulation
TEP: Tms-evoked potentials
TMS: Transcranial magnetic stimulation
ToC: Theory of Consciousness
VS/UWS: Vegetative state/unresponsive wakefulness syndrome
WHIM: Wessex head injury matrix
WNSSP: Western neuro sensory stimulation profile

## References

[1] Jennett B, Plum F. Persistent vegetative state after brain damage. a syndrome in search of a name. Lancet. 1972;1(7753):734–7. 4111204 10.1016/s0140-6736(72)90242-5

[2] Giacino JT, Ashwal S, Childs N, Cranford R, Jennett B, Katz DI, et al. The minimally conscious state: definition and diagnostic criteria. Neurology. 2002;58(3):349–53. 11839831 10.1212/wnl.58.3.349

[3] Zhao J. Disorders of consciousness in China. Neurosci Bull. 2018;34(4):605–14. 30039244 10.1007/s12264-018-0263-1 PMC6060217

[4] Chen WG, Li R, Zhang Y, Hao JH, Du JB, Guo AS, et al. Recovery from prolonged disorders of consciousness: a dual-center prospective cohort study in China. World J Clin Cases. 2020;8(12):2520–9. 32607329 10.12998/wjcc.v8.i12.2520 PMC7322439

[5] Thibaut A, Schiff N, Giacino J, Laureys S, Gosseries O. Therapeutic interventions in patients with prolonged disorders of consciousness. Lancet Neurol. 2019;18(6):600–14. 31003899 10.1016/S1474-4422(19)30031-6

[6] Schnakers C, Laureys S. Coma and disorders of consciousness. London: Springer Verlag London Ltd.; 2014. 10.1007/978-1-4471-2440-5.

[7] Steppacher I, Kaps M, Kissler J. Will time heal? A long-term follow-up of severe disorders of consciousness. Ann Clin Transl Neurol. 2014;1(6):401–8. 25356410 10.1002/acn3.63 PMC4184668

[8] Seel RT, Douglas J, Dennison AC, Heaner S, Farris K, Rogers C. Specialized early treatment for persons with disorders of consciousness: program components and outcomes. Arch Phys Med Rehabil. 2013;94(10):1908–23. 23732166 10.1016/j.apmr.2012.11.052

[9] Schiff ND, Giacino JT, Kalmar K, Victor JD, Baker K, Gerber M, et al. Behavioural improvements with thalamic stimulation after severe traumatic brain injury. Nature. 2007;448(7153):600–3. 17671503 10.1038/nature06041

[10] Edlow BL, Claassen J, Schiff ND, Greer DM. Recovery from disorders of consciousness: mechanisms, prognosis and emerging therapies. Nat Rev Neurol. 2021;17(3):135–56. 33318675 10.1038/s41582-020-00428-x PMC7734616

[11] Afrasiabi M, Redinbaugh MJ, Phillips JM, Kambi NA, Mohanta S, Raz A, et al. Consciousness depends on integration between parietal cortex, striatum, and thalamus. Cell Syst. 2021;12(4):363-73.e11. 33730543 10.1016/j.cels.2021.02.003 PMC8084606

[12] Moruzzi G, Magoun HW. Brain stem reticular formation and activation of the EEG. Electroencephalogr Clin Neurophysiol. 1949;1(4):455–73. 18421835

[13] McLardy T, Ervin F, Mark V, Scoville W, Sweet W. Attempted inset-electrodes-arousal from traumatic coma: neuropathological findings. Trans Am Neurol Assoc. 1968;93:25–30. 4885742

[14] Hassler R, Ore GD, Dieckmann G, Bricolo A, Dolce G. Behavioural and EEG arousal induced by stimulation of unspecific projection systems in a patient with post-traumatic apallic syndrome. Electroencephalogr Clin Neurophysiol. 1969;27(3):306–10. 4185661 10.1016/0013-4694(69)90060-1

[15] Sturm V, Kühner A, Schmitt HP, Assmus H, Stock G. Chronic electrical stimulation of the thalamic unspecific activating system in a patient with coma due to midbrain and upper brain stem infarction. Acta Neurochir. 1979;47(3–4):235–44. 314229 10.1007/BF01406406

[16] Cao T, He S, Wang L, Chai X, He Q, Liu D, et al. Clinical neuromodulatory effects of deep brain stimulation in disorder of consciousness: a literature review. CNS Neurosci Ther. 2024;30(6): e14559. 38115730 10.1111/cns.14559 PMC11163193

[17] Tsubokawa T, Yamamoto T, Katayama Y, Hirayama T, Maejima S, Moriya T. Deep-brain stimulation in a persistent vegetative state: follow-up results and criteria for selection of candidates. Brain Inj. 1990;4(4):315–27. 2252964 10.3109/02699059009026185

[18] Katayama Y, Tsubokawa T, Yamamoto T, Hirayama T, Miyazaki S, Koyama S. Characterization and modification of brain activity with deep brain stimulation in patients in a persistent vegetative state: pain-related late positive component of cerebral evoked potential. Pacing Clin Electrophysiol. 1991;14(1):116–21. 1705325 10.1111/j.1540-8159.1991.tb04055.x

[19] Hosobuchi Y, Yingling C. The treatment of prolonged coma with neurostimulation. Adv Neurol. 1993;63:247–51. 8279309

[20] Cohadon F, Richer E. Deep cerebral stimulation in patients with post-traumatic vegetative state. 25 cases. Neurochirurgie. 1993;39:281–92. 8065486

[21] Yamamoto T, Kobayashi K, Kasai M, Oshima H, Fukaya C, Katayama Y. DBS therapy for the vegetative state and minimally conscious state. Acta Neurochir Suppl. 2005;93:101–4. 15986737 10.1007/3-211-27577-0_17

[22] Yamamoto T, Katayama Y, Kobayashi K, Oshima H, Fukaya C, Tsubokawa T. Deep brain stimulation for the treatment of vegetative state. Eur J Neurosci. 2010;32(7):1145–51. 21039954 10.1111/j.1460-9568.2010.07412.x

[23] Schiff ND. Recovery of consciousness after brain injury: a mesocircuit hypothesis. Trends Neurosci. 2010;33(1):1–9. 19954851 10.1016/j.tins.2009.11.002 PMC2931585

[24] Wojtecki L, Petri D, Elben S, Hirschmann J, Yelnik J, Eickhoff S, et al. Modulation of central thalamic oscillations during emotional-cognitive processing in chronic disorder of consciousness. Cortex. 2014;60:94–102. 25444578 10.1016/j.cortex.2014.09.007

[25] Magrassi L, Maggioni G, Pistarini C, Di Perri C, Bastianello S, Zippo AG, et al. Results of a prospective study (CATS) on the effects of thalamic stimulation in minimally conscious and vegetative state patients. J Neurosurg. 2016;125(4):972–81. 26745476 10.3171/2015.7.JNS15700

[26] Chudy D, Deletis V, Almahariq F, Marcinkovic P, Skrlin J, Paradzik V. Deep brain stimulation for the early treatment of the minimally conscious state and vegetative state: experience in 14 patients. J Neurosurg. 2018;128(4):1189–98. 28621620 10.3171/2016.10.JNS161071

[27] Lemaire JJ, Sontheimer A, Pereira B, Coste J, Rosenberg S, Sarret C, et al. Deep brain stimulation in five patients with severe disorders of consciousness. Ann Clin Transl Neurol. 2018;5:1372–84. 30480031 10.1002/acn3.648 PMC6243378

[28] Gottshall JL, Adams ZM, Forgacs PB, Schiff ND. Daytime central thalamic deep brain stimulation modulates sleep dynamics in the severely injured brain: mechanistic insights and a novel framework for alpha-delta sleep generation. Front Neurol. 2019;10:20. 30778326 10.3389/fneur.2019.00020 PMC6369150

[29] Raguž M, Predrijevac N, Dlaka D, Oreskovic D, Rotim A, Romic D, et al. Structural changes in brains of patients with disorders of consciousness treated with deep brain stimulation. Sci Rep. 2021;11(1):4401. 33623134 10.1038/s41598-021-83873-y PMC7902623

[30] Arnts H, Tewarie P, van Erp WS, Overbeek BU, Stam CJ, Lavrijsen J, et al. Clinical and neurophysiological effects of central thalamic deep brain stimulation in the minimally conscious state after severe brain injury. Sci Rep. 2022;12(1):12932. 35902627 10.1038/s41598-022-16470-2 PMC9334292

[31] Dang Y, Wang Y, Xia X, Yang Y, Bai Y, Zhang J, et al. Deep brain stimulation improves electroencephalogram functional connectivity of patients with minimally conscious state. CNS Neurosci Ther. 2023;29(1):344–53. 36377433 10.1111/cns.14009 PMC9804046

[32] Yang Y, He Q, Dang Y, Xia X, Xu X, Chen X, et al. Long-term functional outcomes improved with deep brain stimulation in patients with disorders of consciousness. Stroke Vasc Neurol. 2023;8(5):368–78. 36882201 10.1136/svn-2022-001998 PMC10647871

[33] Shu Z, Wu J, Li H, Liu J, Lu J, Lin J, et al. fNIRS-based functional connectivity signifies recovery in patients with disorders of consciousness after DBS treatment. Clin Neurophysiol. 2023;147:60–8. 36702043 10.1016/j.clinph.2022.12.011

[34] Chudy D, Deletis V, Paradžik V, Dubroja I, Marčinković P, Orešković D, et al. Deep brain stimulation in disorders of consciousness: 10 years of a single center experience. Sci Rep. 2023;13(1):19491. 37945710 10.1038/s41598-023-46300-y PMC10636144

[35] Schiff ND, Giacino JT, Butson CR, Choi EY, Baker JL, Sullivan O, KP, et al. Thalamic deep brain stimulation in traumatic brain injury: a phase 1, randomized feasibility study. Nat Med. 2023;29(12):3162–74. 38049620 10.1038/s41591-023-02638-4 PMC11087147

[36] Tasserie J, Uhrig L, Sitt JD, Manasova D, Dupont M, Dehaene S, et al. Deep brain stimulation of the thalamus restores signatures of consciousness in a nonhuman primate model. Sci Adv. 2022;8(11):eab15547. 10.1126/sciadv.abl5547 PMC8932660 35302854

[37] Schiff ND. Central thalamic deep brain stimulation to support anterior forebrain mesocircuit function in the severely injured brain. J Neural Transm. 2016;123(7):797–806. 27113938 10.1007/s00702-016-1547-0

[38] Kundu B, Brock AA, Englot DJ, Butson CR, Rolston JD. Deep brain stimulation for the treatment of disorders of consciousness and cognition in traumatic brain injury patients: a review. Neurosurg Focus. 2018;45(2):E14. 30064315 10.3171/2018.5.FOCUS18168 PMC6193266

[39] Liu J, Lee HJ, Weitz AJ, Fang Z, Lin P, Choy M, et al. Frequency-selective control of cortical and subcortical networks by central thalamus. Elife. 2015;4: e09215. 26652162 10.7554/eLife.09215 PMC4721962

[40] Lozano AM, Lipsman N, Bergman H, Brown P, Chabardes S, Chang JW, et al. Deep brain stimulation: current challenges and future directions. Nat Rev Neurol. 2019;15(3):148–60. 30683913 10.1038/s41582-018-0128-2 PMC6397644

[41] Herrington TM, Cheng JJ, Eskandar EN. Mechanisms of deep brain stimulation. J Neurophysiol. 2016;115(1):19–38. 26510756 10.1152/jn.00281.2015 PMC4760496

[42] Mashour GA, Pal D, Brown EN. Prefrontal cortex as a key node in arousal circuitry. Trends Neurosci. 2022;45(10):722–32. 35995629 10.1016/j.tins.2022.07.002 PMC9492635

[43] Seth AK, Bayne T. Theories of consciousness. Nat Rev Neurosci. 2022;23(7):439–52. 35505255 10.1038/s41583-022-00587-4

[44] Romo R, Rossi-Pool R. Towards a conscious model of consciousness. Cogn Neuropsychol. 2020;37(3–4):220–3. 32066320 10.1080/02643294.2020.1728241

[45] Fridman EA, Beattie BJ, Broft A, Laureys S, Schiff ND. Regional cerebral metabolic patterns demonstrate the role of anterior forebrain mesocircuit dysfunction in the severely injured brain. Proc Natl Acad Sci U S A. 2014;111(17):6473–8. 24733913 10.1073/pnas.1320969111 PMC4035959

[46] Williams ST, Conte MM, Goldfine AM, Noirhomme Q, Gosseries O, Thonnard M, et al. Common resting brain dynamics indicate a possible mechanism underlying zolpidem response in severe brain injury. Elife. 2013;2: e01157. 24252875 10.7554/eLife.01157 PMC3833342

[47] Lyu D, Naik S, Menon DK, Stamatakis EA. Intrinsic brain dynamics in the default mode network predict involuntary fluctuations of visual awareness. Nat Commun. 2022;13:6923. 36376303 10.1038/s41467-022-34410-6 PMC9663583

[48] Wu X, Zou Q, Hu J, Tang W, Mao Y, Gao L, et al. Intrinsic functional connectivity patterns predict consciousness level and recovery outcome in acquired brain injury. J Neurosci. 2015;35(37):12932–46. 26377477 10.1523/JNEUROSCI.0415-15.2015 PMC4571611

[49] Rosazza C, Andronache A, Sattin D, Bruzzone MG, Marotta G, Nigri A, et al. Multimodal study of default-mode network integrity in disorders of consciousness. Ann Neurol. 2016;79(5):841–53. 26970235 10.1002/ana.24634

[50] Coulborn S, Taylor C, Naci L, Owen AM, Fernández-Espejo D. Disruptions in effective connectivity within and between default mode network and anterior forebrain mesocircuit in prolonged disorders of consciousness. Brain Sci. 2021;11(6):749. 34200092 10.3390/brainsci11060749 PMC8227204

[51] Zhang YS, Takahashi DY, El HA, Liao DA, Ghazanfar AA. Active neural coordination of motor behaviors with internal states. Proc Natl Acad Sci U S A. 2022;119(39): e2201194119. 36122243 10.1073/pnas.2201194119 PMC9522379

[52] Panda R, Thibaut A, Lopez-Gonzalez A, Escrichs A, Bahri MA, Hillebrand A, et al. Disruption in structural-functional network repertoire and time-resolved subcortical fronto-temporoparietal connectivity in disorders of consciousness. Elife 2022; 11. 10.7554/eLife.77462 PMC9385205 35916363

[53] Luppi AI, Mediano P, Rosas FE, Allanson J, Pickard J, Carhart-Harris RL, et al. A synergistic workspace for human consciousness revealed by integrated information decomposition. Elife. 2024;12:RP88173. 39022924 10.7554/eLife.88173 PMC11257694

[54] Thibaut A, Bruno MA, Chatelle C, Gosseries O, Vanhaudenhuyse A, Demertzi A, et al. Metabolic activity in external and internal awareness networks in severely brain-damaged patients. J Rehabil Med. 2012;44(6):487–94. 22366927 10.2340/16501977-0940

[55] Redinbaugh MJ, Phillips JM, Kambi NA, Mohanta S, Andryk S, Dooley GL, et al. Thalamus modulates consciousness via layer-specific control of cortex. Neuron. 2020;106(1):66-75.e12. 32053769 10.1016/j.neuron.2020.01.005 PMC7243351

[56] Schiff ND. Central lateral thalamic nucleus stimulation awakens cortex via modulation of cross-regional, laminar-specific activity during general anesthesia. Neuron. 2020;106(1):1–3. 32272061 10.1016/j.neuron.2020.02.016

[57] Schiff ND. Mesocircuit mechanisms in the diagnosis and treatment of disorders of consciousness. Presse Med. 2023;52(2): 104161. 36563999 10.1016/j.lpm.2022.104161

[58] Bodien YG, Vora I, Barra A, Chiang K, Chatelle C, Goostrey K, et al. Feasibility and validity of the coma recovery scale-revised for accelerated standardized testing: a practical assessment tool for detecting consciousness in the intensive care unit. Ann Neurol. 2023;94:919–24. 37488068 10.1002/ana.26740 PMC10701693

[59] Ahmadi S, Sarveazad A, Babahajian A, Ahmadzadeh K, Yousefifard M. Comparison of glasgow coma scale and full outline of unresponsiveness score for prediction of in-hospital mortality in traumatic brain injury patients: a systematic review and meta-analysis. Eur J Trauma Emerg Surg. 2023;49(4):1693–706. 36152069 10.1007/s00068-022-02111-w

[60] Rappaport M, Dougherty AM, Kelting DL. Evaluation of coma and vegetative states. Arch Phys Med Rehabil. 1992;73(7):628–34. 1622317

[61] Onami S, Tran D, Koh-Pham C, Shih W, Chi B, Peng J, et al. Coma recovery scale-revised predicts disability rating scale in acute rehabilitation of severe traumatic brain injury. Arch Phys Med Rehabil. 2023;104(7):1054–61. 36736600 10.1016/j.apmr.2023.01.007 PMC10404472

[62] Cortese MD, Arcuri F, Vatrano M, Pioggia G, Cerasa A, Raso MG, et al. Wessex head injury matrix in patients with prolonged disorders of consciousness: a reliability study. Biomedicines. 2023;12(1):82. 38255189 10.3390/biomedicines12010082 PMC10813453

[63] Ansell BJ, Keenan JE. The Western Neuro Sensory Stimulation Profile: a tool for assessing slow-to-recover head-injured patients. Arch Phys Med Rehabil. 1989;70(2):104–8. 2916926

[64] de Conceição TL, Blacker D, Campos C, Garrett C, Duport S, Rocha NB. Repeated clinical assessment using sensory modality assessment and rehabilitation technique for diagnosis in prolonged disorders of consciousness. Front Hum Neurosci. 2021;15: 728637. 34924975 10.3389/fnhum.2021.728637 PMC8671934

[65] Kavusipur S, Rojhani Shirazi Z, Ardekani Z, Omidi S. Prediction of consciousness recovery in coma after traumatic brain injury by disorder of consciousness scale (DOCS). Bull Emerg Trauma. 2013;1(2):86–9. 27162830 PMC4771229

[66] Adams ZM, Forgacs PB, Conte MM, Nauvel TJ, Drover JD, Schiff ND. Late and progressive alterations of sleep dynamics following central thalamic deep brain stimulation (CT-DBS) in chronic minimally conscious state. Clin Neurophysiol. 2016;127(9):3086–92. 27472544 10.1016/j.clinph.2016.06.028 PMC5582539

[67] Curley WH, Bodien YG, Zhou DW, Conte MM, Foulkes AS, Giacino JT, et al. Electrophysiological correlates of thalamocortical function in acute severe traumatic brain injury. Cortex. 2022;152:136–52. 35569326 10.1016/j.cortex.2022.04.007 PMC9759728

[68] Claassen J, Doyle K, Matory A, Couch C, Burger KM, Velazquez A, et al. Detection of brain activation in unresponsive patients with acute brain injury. N Engl J Med. 2019;380(26):2497–505. 31242361 10.1056/NEJMoa1812757

[69] Zhang H, Zhou Q, Chen H, Hu X, Li W, Bai Y, et al. The applied principles of EEG analysis methods in neuroscience and clinical neurology. Mil Med Res. 2023;10(1):67. 38115158 10.1186/s40779-023-00502-7 PMC10729551

[70] Benghanem S, Pruvost-Robieux E, Bouchereau E, Gavaret M, Cariou A. Prognostication after cardiac arrest: how EEG and evoked potentials may improve the challenge. Ann Intensive Care. 2022;12(1):111. 36480063 10.1186/s13613-022-01083-9 PMC9732180

[71] Schiff ND. Mesocircuit mechanisms underlying recovery of consciousness following severe brain injuries: model and predictions. Cham: Springer International Publishing; 2016. p. 195–204.

[72] Steriade M. Grouping of brain rhythms in corticothalamic systems. Neuroscience. 2006;137(4):1087–106. 16343791 10.1016/j.neuroscience.2005.10.029

[73] Sanchez-Vives MV, Massimini M, Mattia M. Shaping the default activity pattern of the cortical network. Neuron. 2017;94(5):993–1001. 28595056 10.1016/j.neuron.2017.05.015

[74] Schiff ND, Nauvel T, Victor JD. Large-scale brain dynamics in disorders of consciousness. Curr Opin Neurobiol. 2014;25:7–14. 24709594 10.1016/j.conb.2013.10.007 PMC3980494

[75] Giacino JT, Fins JJ, Laureys S, Schiff ND. Disorders of consciousness after acquired brain injury: the state of the science. Nat Rev Neurol. 2014;10(2):99–114. 24468878 10.1038/nrneurol.2013.279

[76] Drover JD, Schiff ND, Victor JD. Dynamics of coupled thalamocortical modules. J Comput Neurosci. 2010;28(3):605–16. 20490643 10.1007/s10827-010-0244-5

[77] Forgacs PB, Conte MM, Fridman EA, Voss HU, Victor JD, Schiff ND. Preservation of electroencephalographic organization in patients with impaired consciousness and imaging-based evidence of command-following. Ann Neurol. 2014;76(6):869–79. 25270034 10.1002/ana.24283 PMC4354809

[78] Estraneo A, Loreto V, Guarino I, Boemia V, Paone G, Moretta P, et al. Standard EEG in diagnostic process of prolonged disorders of consciousness. Clin Neurophysiol. 2016;127(6):2379–85. 27178856 10.1016/j.clinph.2016.03.021

[79] Kondziella D, Bender A, Diserens K, van Erp W, Estraneo A, Formisano R, et al. European academy of neurology guideline on the diagnosis of coma and other disorders of consciousness. Eur J Neurol. 2020;27(5):741–56. 32090418 10.1111/ene.14151

[80] Bagnato S, Boccagni C, Prestandrea C. Prognostic value of standard EEG in traumatic and non-traumatic disorders of consciousness following coma. Clin Neurophysiol. 2010;121(3):274–80. 20005157 10.1016/j.clinph.2009.11.008

[81] Bagnato S, Boccagni C. EEG predictors of outcome in patients with disorders of consciousness admitted for intensive rehabilitation. Clin Neurophysiol. 2015;126(5):959–66. 25238957 10.1016/j.clinph.2014.08.005

[82] Chennu S, Annen J, Wannez S, Thibaut A, Chatelle C, Cassol H, et al. Brain networks predict metabolism, diagnosis and prognosis at the bedside in disorders of consciousness. Brain. 2017;140(8):2120–32. 28666351 10.1093/brain/awx163

[83] King JR, Sitt JD, Faugeras F, Rohaut B, El KI, Cohen L, et al. Information sharing in the brain indexes consciousness in noncommunicative patients. Curr Biol. 2013;23(19):1914–9. 24076243 10.1016/j.cub.2013.07.075 PMC5635964

[84] Thibaut A, Bruno MA, Ledoux D, Demertzi A, Laureys S. tDCS in patients with disorders of consciousness: sham-controlled randomized double-blind study. Neurology. 2014;82(13):1112–8. 24574549 10.1212/WNL.0000000000000260

[85] Lechinger J, Bothe K, Pichler G, Michitsch G, Donis J, Klimesch W, et al. CRS-R score in disorders of consciousness is strongly related to spectral EEG at rest. J Neurol. 2013;260(9):2348–56. 23765089 10.1007/s00415-013-6982-3

[86] Williams A, Zeng Y, Li Z, Thakor N, Geocadin RG, Bronder J, et al. Quantitative assessment of electroencephalogram reactivity in comatose patients on extracorporeal membrane oxygenation. Int J Neural Syst. 2022;32:2250025. 35443895 10.1142/S0129065722500253 PMC9436243

[87] Ma X, Qi Y, Xu C, Weng Y, Yu J, Sun X, et al. How well do neural signatures of resting-state EEG detect consciousness? A large-scale clinical study. Hum Brain Mapp. 2024;45: e26586. 38433651 10.1002/hbm.26586 PMC10910334

[88] Sitt JD, King J, Karoui IE, Rohaut B, Faugeras F, Gramfort A, et al. Large scale screening of neural signatures of consciousness in patients in a vegetative or minimally conscious state. Brain. 2014;137(8):2258–70. 24919971 10.1093/brain/awu141 PMC4610185

[89] Nelson DV, Esty ML. Neurotherapy for chronic headache following traumatic brain injury. Mil Med Res. 2015;2:22. 26328060 10.1186/s40779-015-0049-y PMC4553922

[90] Zheng R, Qi Z, Wang Z, Xu Z, Wu X, Mao Y. Clinical decision on disorders of consciousness after acquired brain injury: stepping forward. Neurosci Bull. 2023;39(1):138–62. 35804219 10.1007/s12264-022-00909-7 PMC9849546

[91] Stefan S, Schorr B, Lopez-Rolon A, Kolassa IT, Shock JP, Rosenfelder M, et al. Consciousness indexing and outcome prediction with resting-state EEG in severe disorders of consciousness. Brain Topogr. 2018;31(5):848–62. 29666960 10.1007/s10548-018-0643-x

[92] Noirhomme Q, Brecheisen R, Lesenfants D, Antonopoulos G, Laureys S. “Look at my classifier’s result”: disentangling unresponsive from (minimally) conscious patients. Neuroimage. 2017;145:288–303. 26690804 10.1016/j.neuroimage.2015.12.006

[93] Mikołajewska E, Mikołajewski D. Non-invasive EEG-based brain-computer interfaces in patients with disorders of consciousness. Mil Med Res. 2014;1:14. 26056608 10.1186/2054-9369-1-14 PMC4459059

[94] Pan J, Xie Q, Qin P, Chen Y, He Y, Huang H, et al. Prognosis for patients with cognitive motor dissociation identified by brain-computer interface. Brain. 2020;143(4):1177–89. 32101603 10.1093/brain/awaa026 PMC7174053

[95] Shao H, Deng W, Du R, Zhao Y, Jin D, Wei Y. Mismatch negativity and P300 in the diagnosis and prognostic assessment of coma and other disorders of consciousness. Neurocrit Care 2024. 10.1007/s12028-024-02058-3 39043983

[96] Lindenbaum L, Steppacher I, Mehlmann A, Kissler JM. The effect of neural pre-stimulus oscillations on post-stimulus somatosensory event-related potentials in disorders of consciousness. Front Neurosci. 2023;17:1179228. 37360157 10.3389/fnins.2023.1179228 PMC10287968

[97] Aellen FM, Alnes SL, Loosli F, Rossetti AO, Zubler F, De Lucia M, et al. Auditory stimulation and deep learning predict awakening from coma after cardiac arrest. Brain. 2023;146(2):778–88. 36637902 10.1093/brain/awac340 PMC9924902

[98] Herrera-Diaz A, Boshra R, Tavakoli P, Lin CA, Pajankar N, Bagheri E, et al. Tracking auditory mismatch negativity responses during full conscious state and coma. Front Neurol. 2023;14:1111691. 36970526 10.3389/fneur.2023.1111691 PMC10036371

[99] Daltrozzo J, Wioland N, Mutschler V, Kotchoubey B. Predicting coma and other low responsive patients outcome using event-related brain potentials: a meta-analysis. Clin Neurophysiol. 2007;118(3):606–14. 17208048 10.1016/j.clinph.2006.11.019

[100] Beukema S, Gonzalez-Lara LE, Finoia P, Kamau E, Allanson J, Chennu S, et al. A hierarchy of event-related potential markers of auditory processing in disorders of consciousness. Neuroimage Clin. 2016;12:359–71. 27595064 10.1016/j.nicl.2016.08.003 PMC4995605

[101] Gui P, Jiang Y, Zang D, Qi Z, Tan J, Tanigawa H, et al. Assessing the depth of language processing in patients with disorders of consciousness. Nat Neurosci. 2020;23(6):761–70. 32451482 10.1038/s41593-020-0639-1

[102] Sergent C, Faugeras F, Rohaut B, Perrin F, Valente M, Tallon-Baudry C, et al. Multidimensional cognitive evaluation of patients with disorders of consciousness using EEG: a proof of concept study. Neuroimage Clin. 2016;13:455–69. 28116238 10.1016/j.nicl.2016.12.004 PMC5233797

[103] Spampinato DA, Ibanez J, Rocchi L, Rothwell J. Motor potentials evoked by transcranial magnetic stimulation: interpreting a simple measure of a complex system. J Physiol. 2023;601(14):2827–51. 37254441 10.1113/JP281885 PMC10952180

[104] Rosanova M, Gosseries O, Casarotto S, Boly M, Casali AG, Bruno MA, et al. Recovery of cortical effective connectivity and recovery of consciousness in vegetative patients. Brain. 2012;135:1308–20. 22226806 10.1093/brain/awr340 PMC3326248

[105] Ragazzoni A, Pirulli C, Veniero D, Feurra M, Cincotta M, Giovannelli F, et al. Vegetative versus minimally conscious states: a study using TMS-EEG, sensory and event-related potentials. PLoS ONE. 2013;8(2): e57069. 23460826 10.1371/journal.pone.0057069 PMC3584112

[106] Casali AG, Gosseries O, Rosanova M, Boly M, Sarasso S, Casali KR, et al. A theoretically based index of consciousness independent of sensory processing and behavior. Sci Transl Med. 2013;5(198):19ra8105. 10.1126/scitranslmed.3006294 23946194

[107] Casarotto S, Comanducci A, Rosanova M, Sarasso S, Fecchio M, Napolitani M, et al. Stratification of unresponsive patients by an independently validated index of brain complexity. Ann Neurol. 2016;80(5):718–29. 27717082 10.1002/ana.24779 PMC5132045

[108] Ke XY, Hou W, Huang Q, Hou X, Bao XY, Kong WX, et al. Advances in electrical impedance tomography-based brain imaging. Mil Med Res. 2022;9(1):10. 35227324 10.1186/s40779-022-00370-7 PMC8883715

[109] Annen J, Frasso G, Crone JS, Heine L, Di Perri C, Martial C, et al. Regional brain volumetry and brain function in severely brain-injured patients. Ann Neurol. 2018;83(4):842–53. 29572926 10.1002/ana.25214

[110] Wang L, Yang Y, Chen S, Ge M, He J, Yang Z, et al. White matter integrity correlates with residual consciousness in patients with severe brain injury. Brain Imaging Behav. 2018;12(6):1669–77. 29362992 10.1007/s11682-018-9832-1

[111] Weng L, Xie Q, Zhao L, Zhang R, Ma Q, Wang J, et al. Abnormal structural connectivity between the basal ganglia, thalamus, and frontal cortex in patients with disorders of consciousness. Cortex. 2017;90:71–87. 28365490 10.1016/j.cortex.2017.02.011

[112] Ferraro S, Nigri A, Nava S, Rosazza C, Sattin D, Sebastiano DR, et al. Interhemispherical anatomical disconnection in disorders of consciousness patients. J Neurotrauma. 2019;36(10):1535–43. 30520674 10.1089/neu.2018.5820

[113] Zheng ZS, Reggente N, Lutkenhoff E, Owen AM, Monti MM. Disentangling disorders of consciousness: insights from diffusion tensor imaging and machine learning. Hum Brain Mapp. 2017;38:431–43. 27622575 10.1002/hbm.23370 PMC6867135

[114] Snider SB, Bodien YG, Bianciardi M, Brown EN, Wu O, Edlow BL. Disruption of the ascending arousal network in acute traumatic disorders of consciousness. Neurology. 2019;93(13):e1281–7. 31484715 10.1212/WNL.0000000000008163 PMC7011864

[115] Keijzer HM, Duering M, Pasternak O, Meijer FJA, Verhulst MMLH, Tonino BAR, et al. Free water corrected diffusion tensor imaging discriminates between good and poor outcomes of comatose patients after cardiac arrest. Eur Radiol. 2023;33:2139–48. 36418623 10.1007/s00330-022-09245-w PMC9935650

[116] Puybasset L, Perlbarg V, Unrug J, Cassereau D, Galanaud D, Torkomian G, et al. Prognostic value of global deep white matter DTI metrics for 1-year outcome prediction in ICU traumatic brain injury patients: an MRI-COMA and CENTER-TBI combined study. Intensive Care Med. 2022;48:201–12. 34904191 10.1007/s00134-021-06583-z

[117] Tollard E, Galanaud D, Perlbarg V, Sanchez-Pena P, Le Fur Y, Abdennour L, et al. Experience of diffusion tensor imaging and 1H spectroscopy for outcome prediction in severe traumatic brain injury: preliminary results. Crit Care Med. 2009;37(4):1448–55. 19242330 10.1097/CCM.0b013e31819cf050

[118] Keijzer HM, Lange PAM, Meijer FJA, Tonino BAR, Blans MJ, Klijn CJM, et al. MRI markers of brain network integrity relate to neurological outcome in postanoxic coma. Neuroimage Clin. 2022;36: 103171. 36058165 10.1016/j.nicl.2022.103171 PMC9446009

[119] Monti MM, Vanhaudenhuyse A, Coleman MR, Boly M, Pickard JD, Tshibanda L, et al. Willful modulation of brain activity in disorders of consciousness. N Engl J Med. 2010;362(7):579–89. 20130250 10.1056/NEJMoa0905370

[120] Gibson RM, Fernández-Espejo D, Gonzalez-Lara LE, Kwan BY, Lee DH, Owen AM, et al. Multiple tasks and neuroimaging modalities increase the likelihood of detecting covert awareness in patients with disorders of consciousness. Front Hum Neurosci. 2014;8:950. 25505400 10.3389/fnhum.2014.00950 PMC4244609

[121] Monti MM, Rosenberg M, Finoia P, Kamau E, Pickard JD, Owen AM. Thalamo-frontal connectivity mediates top-down cognitive functions in disorders of consciousness. Neurology. 2015;84(2):167–73. 25480912 10.1212/WNL.0000000000001123 PMC4336082

[122] Di Perri C, Amico E, Heine L, Annen J, Martial C, Larroque SK, et al. Multifaceted brain networks reconfiguration in disorders of consciousness uncovered by co-activation patterns. Hum Brain Mapp. 2018;39(1):89–103. 29024197 10.1002/hbm.23826 PMC6866397

[123] Demertzi A, Tagliazucchi E, Dehaene S, Deco G, Barttfeld P, Raimondo F, et al. Human consciousness is supported by dynamic complex patterns of brain signal coordination. Sci Adv. 2019;5(2):eaat603. 10.1126/sciadv.aat7603 PMC6365115 30775433

[124] Cao B, Chen Y, Yu R, Chen L, Chen P, Weng Y, et al. Abnormal dynamic properties of functional connectivity in disorders of consciousness. Neuroimage Clin. 2019;24: 102071. 31795053 10.1016/j.nicl.2019.102071 PMC6881656

[125] Raguz M, Almahariq F, Predrijevac N, Deletis V, Marcinkovic P, Chudy D. Deep brain stimulation for vegetative and minimal conscious state volumetric analysis in patients after electrode implantation in centromedian parafascicular complex. Stereot Funct Neuros. 2019;97:56.

[126] Farg H, Elnakib A, Gebreil A, Alksas A, van Bogaert E, Mahmoud A, et al. Diagnostic value of PET imaging in clinically unresponsive patients. Br J Radiol. 2024;97(1154):283–91. 38308033 10.1093/bjr/tqad040

[127] He Z, Lu R, Ge J, Guan Y, Chen Y, Liu G, et al. Disorder of consciousness related pattern could distinguish minimally conscious state from unresponsive wakefulness syndrome: a F-18-FDG-PET study. Brain Res Bull. 2024;215: 111023. 38964662 10.1016/j.brainresbull.2024.111023

[128] Bodien YG, Chatelle C, Edlow BL. Functional networks in disorders of consciousness. Semin Neurol. 2017;37(5):485–502. 29207410 10.1055/s-0037-1607310 PMC5884076

[129] Boly M, Faymonville ME, Schnakers C, Peigneux P, Lambermont B, Phillips C, et al. Perception of pain in the minimally conscious state with PET activation: an observational study. Lancet Neurol. 2008;7(11):1013–20. 18835749 10.1016/S1474-4422(08)70219-9

[130] Stender J, Gosseries O, Bruno MA, Charland-Verville V, Vanhaudenhuyse A, Demertzi A, et al. Diagnostic precision of PET imaging and functional MRI in disorders of consciousness: a clinical validation study. Lancet. 2014;384(9942):514–22. 24746174 10.1016/S0140-6736(14)60042-8

[131] Plosnić G, Raguž M, Deletis V, Chudy D. Dysfunctional connectivity as a neurophysiologic mechanism of disorders of consciousness: a systematic review. Front Neurosci. 2023;17:1166187. 37539385 10.3389/fnins.2023.1166187 PMC10394244

[132] Annen J, Frasso G, van der Lande GJM, Bonin EAC, Vitello MM, Panda R, et al. Cerebral electrometabolic coupling in disordered and normal states of consciousness. Cell Rep. 2023;42(8): 112854. 37498745 10.1016/j.celrep.2023.112854

[133] Whyte CJ, Redinbaugh MJ, Shine JM, Saalmann YB. Thalamic contributions to the state and contents of consciousness. Neuron. 2024;112(10):1611–25. 38754373 10.1016/j.neuron.2024.04.019 PMC11537458

[134] Vanhoecke J, Hariz M. Deep brain stimulation for disorders of consciousness: systematic review of cases and ethics. Brain Stimul. 2017;10(6):1013–23. 28966051 10.1016/j.brs.2017.08.006

[135] Rezaei Haddad A, Lythe V, Green AL. Deep brain stimulation for recovery of consciousness in minimally conscious patients after traumatic brain injury: a systematic review. Neuromodulation. 2019;22(4):373–9. 30865342 10.1111/ner.12944

[136] Boeken OJ, Cieslik EC, Langner R, Markett S. Characterizing functional modules in the human thalamus: coactivation-based parcellation and systems-level functional decoding. Brain Struct Funct. 2023;228(8):1811–34. 36547707 10.1007/s00429-022-02603-w PMC10516793

[137] Jankowski MM, Ronnqvist KC, Tsanov M, Vann SD, Wright NF, Erichsen JT, et al. The anterior thalamus provides a subcortical circuit supporting memory and spatial navigation. Front Syst Neurosci. 2013;7:45. 24009563 10.3389/fnsys.2013.00045 PMC3757326

[138] Schreiner T, Kaufmann E, Noachtar S, Mehrkens JH, Staudigl T. The human thalamus orchestrates neocortical oscillations during NREM sleep. Nat Commun. 2022;13:5231. 36064855 10.1038/s41467-022-32840-w PMC9445182

[139] Fan L. Mapping the human brain: What is the next frontier? Innovation (Camb). 2020;2(1): 100073. 34557730 10.1016/j.xinn.2020.100073 PMC8454655

[140] Krauth A, Blanc R, Poveda A, Jeanmonod D, Morel A, Szekely G. A mean three-dimensional atlas of the human thalamus: generation from multiple histological data. Neuroimage. 2010;49(3):2053–62. 19853042 10.1016/j.neuroimage.2009.10.042

[141] Gallay MN, Magara AE, Moser D, Kowalski M, Kaeser M, Jeanmonod D. Magnetic resonance-guided focused ultrasound central lateral thalamotomy against chronic and therapy-resistant neuropathic pain: retrospective long-term follow-up analysis of 63 interventions. J Neurosurg. 2023;139(3):615–24. 36840733 10.3171/2023.1.JNS222879

[142] Goto M, Kamagata K, Andica C, Takabayashi K, Uchida W, Goto T, et al. Deep learning-based hierarchical brain segmentation with preliminary analysis of the repeatability and reproducibility. Magn Reson Med Sci 2024. 10.2463/mrms.mp.2023-0124 PMC12406159 38960679

[143] Iglesias JE, Insausti R, Lerma-Usabiaga G, Bocchetta M, Van Leemput K, Greve DN, et al. A probabilistic atlas of the human thalamic nuclei combining ex vivo MRI and histology. Neuroimage. 2018;183:314–26. 30121337 10.1016/j.neuroimage.2018.08.012 PMC6215335

[144] Middlebrooks EH, Tao S, Zhou X, Greco E, Westerhold EM, Tipton PW, et al. Synthetic inversion image generation using MP2RAGE T1 mapping for surgical targeting in deep brain stimulation and lesioning. Stereotact Funct Neurosurg. 2023;101(5):326–31. 37607507 10.1159/000533259

[145] Traynor CR, Barker GJ, Crum WR, Williams SCR, Richardson MP. Segmentation of the thalamus in MRI based on T1 and T2. Neuroimage. 2011;56(3):939–50. 21310246 10.1016/j.neuroimage.2011.01.083

[146] Solomon E, Lotan E, Zan E, Sodickson DK, Block KT, Chandarana H. MP-RAVE: IR-prepared T_1_ -Weighted Radial Stack-of-Stars 3D GRE imaging with retrospective motion correction. Magn Reson Med. 2023;90(1):202–10. 36763847 10.1002/mrm.29614 PMC10323698

[147] Su JH, Thomas FT, Kasoff WS, Tourdias T, Choi EY, Rutt BK, et al. Thalamus optimized multi atlas segmentation (THOMAS): fast, fully automated segmentation of thalamic nuclei from structural MRI. Neuroimage. 2019;194:272–82. 30894331 10.1016/j.neuroimage.2019.03.021 PMC6536348

[148] Najdenovska E, Tuleasca C, Jorge J, Maeder P, Marques JP, Roine T, et al. Comparison of MRI-based automated segmentation methods and functional neurosurgery targeting with direct visualization of the ventro-intermediate thalamic nucleus at 7T. Sci Rep. 2019;9(1):1119. 30718634 10.1038/s41598-018-37825-8 PMC6361927

[149] Corona V, Lellmann J, Nestor P, Schonlieb CB, Acosta-Cabronero J. A multi-contrast MRI approach to thalamus segmentation. Hum Brain Mapp. 2020;41(8):2104–20. 31957926 10.1002/hbm.24933 PMC7267924

[150] Zhang D, Snyder AZ, Fox MD, Sansbury MW, Shimony JS, Raichle ME. Intrinsic functional relations between human cerebral cortex and thalamus. J Neurophysiol. 2008;100(4):1740–8. 18701759 10.1152/jn.90463.2008 PMC2576214

[151] Kumar VJ, van Oort E, Scheffler K, Beckmann CF, Grodd W. Functional anatomy of the human thalamus at rest. Neuroimage. 2017;147:678–91. 28041978 10.1016/j.neuroimage.2016.12.071

[152] Tian Y, Margulies DS, Breakspear M, Zalesky A. Topographic organization of the human subcortex unveiled with functional connectivity gradients. Nat Neurosci. 2020;23(11):1421–32. 32989295 10.1038/s41593-020-00711-6

[153] Patriat R, Palnitkar T, Chandrasekaran J, Sretavan K, Braun H, Yacoub E, et al. DiMANI: diffusion MRI for anatomical nuclei imaging-application for the direct visualization of thalamic subnuclei. Front Hum Neurosci. 2024;18:1324710. 38439939 10.3389/fnhum.2024.1324710 PMC10910100

[154] Yang Y, Xu H, Deng Z, Cheng W, Zhao X, Wu Y, et al. Functional connectivity and structural changes of thalamic subregions in episodic migraine. J Headache Pain. 2022;23(1):119. 36088305 10.1186/s10194-022-01491-z PMC9463803

[155] Jonasson L, Hagmann P, Pollo C, Bresson X, Richero Wilson C, Meuli R, et al. A level set method for segmentation of the thalamus and its nuclei in DT-MRI. Signal Process. 2007;87(7):309–21.

[156] Ye C, Bogovic JA, Ying SH, Prince JL. Parcellation of the thalamus using diffusion tensor images and a multi-object geometric deformable model. Proc SPIE Int Soc Opt Eng. 2013. 10.1117/12.2006119. 24382992 10.1117/12.2006119 PMC3875234

[157] Kumar V, Mang S, Grodd W. Direct diffusion-based parcellation of the human thalamus. Brain Struct Funct. 2015;220(3):1619–35. 24659254 10.1007/s00429-014-0748-2

[158] Battistella G, Najdenovska E, Maeder P, Ghazaleh N, Daducci A, Thiran JP, et al. Robust thalamic nuclei segmentation method based on local diffusion magnetic resonance properties. Brain Struct Funct. 2017;222(5):2203–16. 27888345 10.1007/s00429-016-1336-4 PMC5504280

[159] Gao C, Wu X, Wang Y, Li G, Ma L, Wang C, et al. Prior-guided individualized thalamic parcellation based on local diffusion characteristics. Hum Brain Mapp. 2024;45(4): e26646. 38433705 10.1002/hbm.26646 PMC10910286

[160] Schiff ND. Brain function and responsiveness in disorders of consciousness. In: Sannita WG, editor. Monti MM. Cham: Springer International Publishing; 2016.

[161] Giacino J, Fins JJ, Machado A, Schiff ND. Central thalamic deep brain stimulation to promote recovery from chronic posttraumatic minimally conscious state: challenges and opportunities. Neuromodulation. 2012;15(4):339–49. 22624587 10.1111/j.1525-1403.2012.00458.x

[162] Magrassi L, Zippo AG, Azzalin A, Bastianello S, Imberti R, Biella G. Single unit activities recorded in the thalamus and the overlying parietal cortex of subjects affected by disorders of consciousness. PLoS ONE. 2018;13(11): e0205967. 30403761 10.1371/journal.pone.0205967 PMC6221278

[163] Pastor J, Vega-Zelaya L, Martín-Abad E. (2021) Neurophysiological characterization of posteromedial hypothalamus in anaesthetized patients. Brain Sci 12. 10.3390/brainsci12010043 PMC8773588 35053786

[164] Vega-Zelaya L, Torres CV, Navas M, Pastor J. Neurophysiological characterization of thalamic nuclei in epileptic anaesthetized patients. Brain Sci. 2019;9(11):312. 31703408 10.3390/brainsci9110312 PMC6895797

[165] Warren AEL, Dalic LJ, Thevathasan W, Roten A, Bulluss KJ, Archer J. Targeting the centromedian thalamic nucleus for deep brain stimulation. J Neurol, Neurosurg Psychiatry. 2020;91(4):339–49. 31980515 10.1136/jnnp-2019-322030

[166] Calderon DP, Schiff ND. Objective and graded calibration of recovery of consciousness in experimental models. Curr Opin Neurol. 2021;34(1):142–9. 33278146 10.1097/WCO.0000000000000895 PMC7866679

[167] Llinás R, Urbano FJ, Leznik E, Ramírez RR, van Marle HJF. Rhythmic and dysrhythmic thalamocortical dynamics: GABA systems and the edge effect. Trends Neurosci. 2005;28(6):325–33. 15927689 10.1016/j.tins.2005.04.006

[168] Onofrj M, Russo M, Delli PS, De Gregorio D, Inserra A, Gobbi G, et al. The central role of the thalamus in psychosis, lessons from neurodegenerative diseases and psychedelics. Transl Psychiatry. 2023;13(1):384. 38092757 10.1038/s41398-023-02691-0 PMC10719401

[169] Hô N, Destexhe A. Synaptic background activity enhances the responsiveness of neocortical pyramidal neurons. J Neurophysiol. 2000;84(3):1488–96. 10980021 10.1152/jn.2000.84.3.1488

[170] Lutkenhoff ES, Wright MJ, Shrestha V, Real C, McArthur DL, Buitrago-Blanco M, et al. The subcortical basis of outcome and cognitive impairment in TBI: a longitudinal cohort study. Neurology. 2020;95(17):e2398–408. 32907958 10.1212/WNL.0000000000010825 PMC7682912

[171] Snider SB, Bodien YG, Frau-Pascual A, Bianciardi M, Foulkes AS, Edlow BL. Ascending arousal network connectivity during recovery from traumatic coma. Neuroimage Clin. 2020;28: 102503. 33395992 10.1016/j.nicl.2020.102503 PMC7724378

[172] Zhang J, Zhang H, Yan F, Zhang H, Zhang E, Wang X, et al. Investigating the mechanism and prognosis of patients with disorders of consciousness on the basis of brain networks between the thalamus and whole-brain. Front Neurol. 2022;13: 990686. 36237619 10.3389/fneur.2022.990686 PMC9552841

[173] Forgacs PB, Allen BB, Wu X, Gerber LM, Boddu S, Fakhar M, et al. Corticothalamic connectivity in aneurysmal subarachnoid hemorrhage: relationship with disordered consciousness and clinical outcomes. Neurocrit Care. 2022;36(3):760–71. 34669180 10.1007/s12028-021-01354-6

[174] Forgacs PB, Frey HP, Velazquez A, Thompson S, Brodie D, Moitra V, et al. Dynamic regimes of neocortical activity linked to corticothalamic integrity correlate with outcomes in acute anoxic brain injury after cardiac arrest. Ann Clin Transl Neurol. 2017;4(2):119–29. 28168211 10.1002/acn3.385 PMC5288467

[175] Yang AI, Raghu ALB, Isbaine F, Alwaki A, Gross RE. Sensing with deep brain stimulation device in epilepsy: aperiodic changes in thalamic local field potential during seizures. Epilepsia (Copenhagen). 2023;64(11):3025–35. 10.1111/epi.17758 37607249

[176] Donoghue J, Bastos AM, Yanar J, Kornblith S, Mahnke M, Brown EN, et al. Neural signatures of loss of consciousness and its recovery by thalamic stimulation. bioRxiv. 2019. 10.1101/806687.

[177] Xu J, Galardi MM, Pok B, Patel KK, Zhao CW, Andrews JP, et al. Thalamic stimulation improves postictal cortical arousal and behavior. J Neurosci. 2020;40(38):7343–54. 32826310 10.1523/JNEUROSCI.1370-20.2020 PMC7534908

[178] Huang Y, He J, Green AL, Aziz TZ, Stein JF, Wang S. Characteristics of thalamic local field potentials in patients with disorders of consciousness. Annu Int Conf IEEE Eng Med Biol Soc. 2015;2015:3779–82. 26737116 10.1109/EMBC.2015.7319216

[179] He J, Zhang H, Dang Y, Zhuang Y, Ge Q, Yang Y, et al. Electrophysiological characteristics of CM-Pf in diagnosis and outcome of patients with disorders of consciousness. Brain Stimul. 2023;16(5):1522–32. 37778457 10.1016/j.brs.2023.09.021

[180] Nourski KV, Steinschneider M, Rhone AE, Kawasaki H, Howard MR, Banks MI. Auditory predictive coding across awareness states under anesthesia: an intracranial electrophysiology study. J Neurosci. 2018;38(39):8441–52. 30126970 10.1523/JNEUROSCI.0967-18.2018 PMC6158689

[181] Nourski KV, Steinschneider M, Rhone AE, Krause BM, Mueller RN, Kawasaki H, et al. Cortical responses to vowel sequences in awake and anesthetized states: a human intracranial electrophysiology study. Cereb Cortex. 2021;31(12):5435–48. 34117741 10.1093/cercor/bhab168 PMC8568007

[182] Zhang Z, Huang Y, Chen X, Li J, Yang Y, Lv L, et al. State-specific regulation of electrical stimulation in the intralaminar thalamus of macaque monkeys: network and transcriptional insights into arousal. Adv Sci (Weinh). 2024;11(33): e2402718. 38938001 10.1002/advs.202402718 PMC11434125

[183] Solovey G, Alonso LM, Yanagawa T, Fujii N, Magnasco MO, Cecchi GA, et al. Loss of consciousness is associated with stabilization of cortical activity. J Neurosci. 2015;35(30):10866–77. 26224868 10.1523/JNEUROSCI.4895-14.2015 PMC4518057

[184] Ma L, Liu W, Hudson AE. Propofol anesthesia increases long-range frontoparietal corticocortical interaction in the oculomotor circuit in macaque monkeys. Anesthesiology. 2019;130(4):560–71. 30807382 10.1097/ALN.0000000000002637 PMC6417961

[185] Breshears JD, Roland JL, Sharma M, Gaona CM, Freudenburg ZV, Tempelhoff R, et al. Stable and dynamic cortical electrophysiology of induction and emergence with propofol anesthesia. Proc Natl Acad Sci U S A. 2010;107(49):21170–5. 21078987 10.1073/pnas.1011949107 PMC3000270

[186] Banks MI, Krause BM, Endemann CM, Campbell DI, Kovach CK, Dyken ME, et al. Cortical functional connectivity indexes arousal state during sleep and anesthesia. Neuroimage. 2020;211: 116627. 32045640 10.1016/j.neuroimage.2020.116627 PMC7117963

[187] Bayne T, Hohwy J, Owen AM. Reforming the taxonomy in disorders of consciousness. Ann Neurol. 2017;82(6):866–72. 29091304 10.1002/ana.25088

[188] Timic Stamenic T, Todorovic SM. Thalamic T-type calcium channels as targets for hypnotics and general anesthetics. Int J Mol Sci. 2022;23(4):2349. 35216466 10.3390/ijms23042349 PMC8876360

[189] Crunelli V, David F, Leresche N, Lambert RC. Role for T-type Ca^2+^ channels in sleep waves. Pflugers Arch. 2014;466(4):735–45. 24578015 10.1007/s00424-014-1477-3

[190] Aru J, Suzuki M, Rutiku R, Larkum ME, Bachmann T. Coupling the state and contents of consciousness. Front Syst Neurosci. 2019;13:43. 31543762 10.3389/fnsys.2019.00043 PMC6729974

[191] Suzuki M, Larkum ME. General anesthesia decouples cortical pyramidal neurons. Cell. 2020;180(4):666-76.e13. 32084339 10.1016/j.cell.2020.01.024

[192] Stender J, Mortensen KN, Thibaut A, Darkner S, Laureys S, Gjedde A, et al. The minimal energetic requirement of sustained awareness after brain injury. Curr Biol. 2016;26(11):1494–9. 27238279 10.1016/j.cub.2016.04.024

[193] Buccellato A, Zang D, Zilio F, Gomez-Pilar J, Wang Z, Qi Z, et al. Disrupted relationship between intrinsic neural timescales and alpha peak frequency during unconscious states—a high-density EEG study. Neuroimage. 2023;265: 119802. 36503159 10.1016/j.neuroimage.2022.119802

[194] Wolff A, Berberian N, Golesorkhi M, Gomez-Pilar J, Zilio F, Northoff G. Intrinsic neural timescales: temporal integration and segregation. Trends Cogn Sci. 2022;26(2):159–73. 34991988 10.1016/j.tics.2021.11.007

[195] Zilio F, Gomez-Pilar J, Chaudhary U, Fogel S, Fomina T, Synofzik M, et al. Altered brain dynamics index levels of arousal in complete locked-in syndrome. Commun Biol. 2023;6(1):757. 37474587 10.1038/s42003-023-05109-1 PMC10359418

[196] Silva LR, Amitai Y, Connors BW. Intrinsic oscillations of neocortex generated by layer 5 pyramidal neurons. Science. 1991;251(4992):432–5. 1824881 10.1126/science.1824881

[197] Bick C, Goodfellow M, Laing CR, Martens EA. Understanding the dynamics of biological and neural oscillator networks through exact mean-field reductions: a review. J Math Neurosci. 2020;10(1):9. 32462281 10.1186/s13408-020-00086-9 PMC7253574

[198] Qin P, Wu X, Huang Z, Duncan NW, Tang W, Wolff A, et al. How are different neural networks related to consciousness? Ann Neurol. 2015;78(4):594–605. 26290126 10.1002/ana.24479

